# Current Principles, Challenges, and New Metrics in pH-Responsive Drug Delivery Systems for Systemic Cancer Therapy

**DOI:** 10.3390/pharmaceutics15051566

**Published:** 2023-05-22

**Authors:** Roman A. Verkhovskii, Alexey N. Ivanov, Ekaterina V. Lengert, Ksenia A. Tulyakova, Natalia Yu. Shilyagina, Alexey V. Ermakov

**Affiliations:** 1 Science Medical Center, Saratov State University, 83 Astrakhanskaya Str., 410012 Saratov, Russia; r.a.verhovskiy@mail.ru; 2 Central Research Laboratory, Saratov State Medical University of V. I. Razumovsky, Ministry of Health of the Russian Federation, 410012 Saratov, Russia; lex558452@gmail.com (A.N.I.); lengert_e_v@staff.sechenov.ru (E.V.L.); 3 Institute of Molecular Theranostics, I. M. Sechenov First Moscow State Medical University, 8 Trubetskaya Str., 119991 Moscow, Russia; 4 Institute of Biology and Biomedicine, Lobachevsky State University of Nizhny Novgorod, 23 Gagarin Ave., 603950 Nizhny Novgorod, Russia; tulyakova.ksenia@gmail.com

**Keywords:** cancer therapy, nanomedicine, drug delivery, pH-responsiveness, EPR, intratumoral delivery, intracellular delivery, nanoparticles, metal–organic frameworks

## Abstract

The paradigm of drug delivery via particulate formulations is one of the leading ideas that enable overcoming limitations of traditional chemotherapeutic agents. The trend toward more complex multifunctional drug carriers is well-traced in the literature. Nowadays, the prospectiveness of stimuli-responsive systems capable of controlled cargo release in the lesion nidus is widely accepted. Both endogenous and exogenous stimuli are employed for this purpose; however, endogenous pH is the most common trigger. Unfortunately, scientists encounter multiple challenges on the way to the implementation of this idea related to the vehicles’ accumulation in off-target tissues, their immunogenicity, the complexity of drug delivery to intracellular targets, and finally, the difficulties in the fabrication of carriers matching all imposed requirements. Here, we discuss fundamental strategies for pH-responsive drug delivery, as well as limitations related to such carriers’ application, and reveal the main problems, weaknesses, and reasons for poor clinical results. Moreover, we attempted to formulate the profiles of an “ideal” drug carrier in the frame of different strategies drawing on the example of metal-comprising materials and considered recently published studies through the lens of these profiles. We believe that this approach will facilitate the formulation of the main challenges facing researchers and the identification of the most promising trends in technology development.

## 1. Introduction

To date, the development of new methods and improvement of already existing methods for cancer therapy occupy a large place in biomedical sciences. However, despite significant advances in both clinical and laboratory research, we are still far from a breakthrough in this field [1,2,3,4]. Research in this area goes along various vectors, from trying to achieve a fundamental understanding of disease mechanisms and developing new drugs to “programming” the body to fight the disease [5,6,7,8,9]. Enormous efforts are devoted to the development of drug delivery systems (DDSs), which, in their turn, occasionally represent a bigger threat to the organism than the drugs themselves. Nevertheless, such systems enable circumventing a range of shortcomings of traditional therapeutic agents, including low bioavailability, susceptibility to the aggressive influence of surrounding media, accelerated blood clearance, and off-target toxicity [10,11,12].

The application of such DDSs for drug delivery through the systemic administration produces new challenges in therapy which are often difficult to predict in advance. Various factors affect the behavior of carriers in the body. Multiple studies have shown that the nature, size, surface potential, and other characteristics of vehicles fundamentally affect their biodistribution, cell interaction, circulation time, and hydrodynamics in the blood flow [13,14,15,16]. Often, carriers’ features providing the effective administration procedure, such as prolonged circulation in the blood flow and low immunogenicity, impede the other desirable features of the DDS, such as a high internalization efficiency by cancer cells and drug release. In this regard, researchers have to take into consideration a huge number of parameters in order to obtain the optimal carrier for a particular task. In this way, the drug delivery paradigm has been applied to encapsulate all sorts of drugs, from cytostatics to photodynamic agents [17,18,19,20,21]. Understanding drug bioavailability issues and fundamental disease mechanisms is driving researchers to develop increasingly complex DDSs designed to provide enhanced drug stability, prolonged circulation time, tolerability, retention in lesion area, increased internalization by a specific cell types, controlled release profile, responsiveness to a specific endogenous and exogenous triggers (IR radiation, magnetic field, electric field, ultrasound, temperature, pH, and enzymes), and others [22,23,24,25].

**Figure 1:**
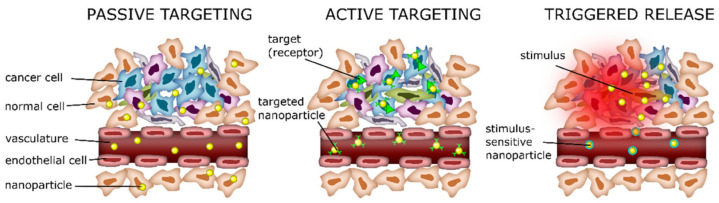
Comparison of passive targeting, active targeting, and triggered release of nanodrugs. Reprinted with permission from [32]. Copyright 2021, MDPI.

In general, the targeting of drug-loaded carriers to tumors in the organism can be divided into active and passive (Figure 1). The passive approach is based on the accumulation of the carriers in pathological sites with affected vasculature, such as tumors, inflammations, and infarcted areas [26]. In this case, drug carriers are supposed to circulate in the blood as long as possible to increase the probability of their accumulation in pathological sites via the enhanced permeability and retention (EPR) effect. Active targeting employs a modification of carriers with specific ligands or different nanostructures possessing certain physical properties, which ensure a specific recognition and bind of DDS with pathological cells [27,28], promotion of their uptake [29], or manipulation over their distribution and drug release by external stimuli such as a magnetic field [30,31]. Usually, researchers employ a hybrid approach when EPR-mediated accumulation promotes the delivery of actively targeted carriers into poorly accessible sites of interest.

Current trends in the field of the DDSs development for chemotherapeutic compounds’ delivery suggest the release of encapsulated substances in response to endogenous or exogenous stimuli in addition to the passive targeting by EPR effect. Thus, the application of thermosensitive liposomes for targeted drug delivery showed up to a 17-times increase in drug concentration in the tumor area compared with the free form [33]. However, this method is applicable only to the treatment of surgically accessible tumors and not to systemic therapy of metastatic disease, which is of paramount importance for cancer therapy. Other studies suggest the use of non-invasive methods for the carriers’ activation and the induction of therapeutic agents’ release by exposure to external physical fields, including magnetic ones, ultrasound, and infrared radiation [22,23,24,31,34,35,36,37]. Moreover, a promising strategy is the usage of the tumor microenvironment’s features, including reduced pH of the tumor milieu (pH in the range from 6.3 to 7), if compared with the pH in normal tissues (7.35–7.45). In this regard, the development of pH-responsive delivery systems that release cargo in response to slightly acidic pH opens up new venues for the treatment of poorly accessible tumors [38,39].

In general, the pH is a rigid biological constant in the human body. However, the pH in soft tissues may differ from that in blood plasma depending on the metabolic activity of cells and surrounding conditions, which makes the usage of pH-sensitive systems for the targeted drug delivery possible. It is known that tumor cells are characterized by increased metabolic activity, which ensures a high proliferation rate. The energy needs of tumor cells are provided mainly by anaerobic glucose metabolism, especially in conditions of poor blood supply and oxygen deprivation, which leads to the accumulation of products of incomplete glucose oxidation [40]. As a result of the active metabolism of cancer cells, the tumor microenvironment has a higher acidity and reduced pH compared with normal tissues, thus making the application of pH-responsive biomaterials highly prospective for targeted drug delivery to malignant neoplasms [41].

The use of pH as an endogenous trigger for the release of active components by stimulus-responsive carriers has a number of advantages, including wide applicability and the absence of the demand for external triggers. At the same time, authors indicate the low accuracy of DDSs delivery to the tumor area, accompanied by drug release in non-targeted sites of the body, as the main disadvantage of pH as triggering stimuli, and this is due to the possibility of shifts in acid–base homeostasis in tissues caused by a wide range of physiological and pathological (inflammatory changes) reasons [42]. Since multiple recently published reviews were dedicated to synthesis strategies and the classification of pH-responsive DDSs [43,44,45,46], here, we focused on the current paradigm and fundamentals of the drug delivery approach based on pH-responsive materials. For this purpose, we discussed the difficulties that researchers face in implementing this idea and the potential solutions to overcome them. In this frame, we discuss a variety of functions drug carriers have to implement to provide a targeted therapeutic effect. Furthermore, we introduced new metrics to compare different DDSs’ configurations aimed at revealing the profile of an “ideal” pH-responsive drug carrier by using the example of metal-comprising carriers. Finally, we overviewed recently published results of animal experiments and clinical studies and considered their results through the prism of carriers’ functionality.

## 2. Principles of pH-Responsive Drug Delivery

In the last few decades, the paradigm of drug carriers’ usage to overcome the non-specific distribution of therapeutic agents in the body, including chemotherapeutic substances that exert severe toxic stress on healthy tissues, has been actively developed. One of the main pillars of this paradigm is the increased or even selective accumulation of DDSs carrying therapeutic agents in tumor interstitium harnessing the differences between normal and cancer tissues properties. Thus, structural features of tumors, such as hypervascularization, vascular pathologies, and impaired functionality of lymphatic drainage, can be utilized to differentiate tumors from healthy tissues and selectively accumulate drug carriers. In particular, tumor-surrounding vessels are characterized by defects in the endothelial layer lining the blood vessel wall, represented by wide fenestrations (up to several microns) and other features that lead to an increase in the permeability of this barrier for small objects, making the effective extravasation of nanosized carriers from the bloodstream to tumor interstitium possible [47,48,49]. Methods of selective therapy via the systemic administration of therapeutic agents based on increased permeability of the tumor vessels’ wall, known under the general name of the EPR effect, have become widespread and have inspired the creation of a large number of vehicles proposed for the delivery of chemotherapeutic agents [50,51]. In summary, the EPR effect implies the extravasation of nanosized drug carriers through endothelial fenestra and their retention in the interstitial volume of the tumor due to dysfunctional lymphatic drainage.

### 2.1. Design of Drug Carriers for pH-Responsive Delivery

The encapsulation of therapeutic compounds in micro-, submicro-, and nanosized carriers has proven to be a promising approach to improve the therapeutic index of anticancer pharmaceuticals [52]. This approach has changed the paradigm of cancer treatment, setting off impressive developments over the past four decades. Modern carriers can serve for multiple issues, including the protection of a therapeutic cargo from degradation in an aggressive extracellular environment, its delivery to the tumor, prevention of chemotherapeutic drug penetration into healthy tissue, and regulation over its release in the region of interest. The last characteristic is one of the most important for DDSs applied for cancer treatment since the success of the targeted therapy via drug-loaded carriers directly depends on their capability to release the cargo precisely in the desired area, specifically after their extravasation into the tumor. In this regard, both methods of passive drug release and approaches to the active triggering of this process are investigated. One of the most common factors used for passive cargo release is pH difference. Current approaches for targeted therapy in oncology mostly employ precise molecular targets, such as specific receptors [53,54], microRNAs [55,56], or the ubiquitin–proteasome pathway [57,58]. However, the acidic pH in extracellular tumor tissues is a common phenotype across a wide range of cancer types that makes it a promising feature for targeted drug delivery [59]. Today, the dominant concept in pH-responsive drug delivery systems is to provide cargo release at the acidic pH of tumor parenchyma. The scientific community suggests different pH values that should trigger cargo release to provide effective therapy, but an average value of 5.7 can be estimated over the literature, as is shown below.

There are a few strategies applied for the design of pH-responsive DDSs, including degradation strategy, gatekeeper strategy, and bond cleavage strategy [60,61,62]. The first one is the widest employed strategy which supposes the complete degradation of drug carriers in response to an acidic microenvironment. A wide variety of materials and mechanisms are shown to induce the breaking down of drug carriers under an acidic tumor microenvironment, resulting in site-specific drug release [63]. Meanwhile, mostly polymeric carriers undergo swelling in dependence on the microenvironment pH, leading to changes in their porosity and chemical bounds [64,65]. The gatekeeper strategy employs an on-demand approach via core–shell structured carriers. Such systems mainly consist of mesoporous materials as a core for drug encapsulation and pH-responsive coating on the surface acting as a «gatekeeper» to provide controlled release [66,67]. The bond cleavage strategy employs acid-labile bonds between DDS surface and drug as loading mechanism, followed by hydrolysis of these bonds to provide drug release in response to an acidic microenvironment [68].

Besides the capability of DDSs to accurately unleash the drug in the tumor interstitium, the systemically administered vehicles are required to provide a range of other features, such as the ability to be delivered with blood flow to the neoplasm to ensure successful cancer treatment. In general, the favored drug carrier size is under 200 nm to provide higher specific surface areas, appropriate circulation time, penetration into tumor tissues, and improved potential for internalization by cancer cells [69,70,71]. However, the high internalization potential of particles leads to their enhanced entrapment by phagocytes, while DDSs should avoid extravasation by the reticuloendothelial system (RES) and kidney to prolong their residence in the circulatory system. This fact makes a negative surface charge an important requirement, as it helps to reduce interactions with negatively charged cells in the blood flow [72].

**Figure 2:**
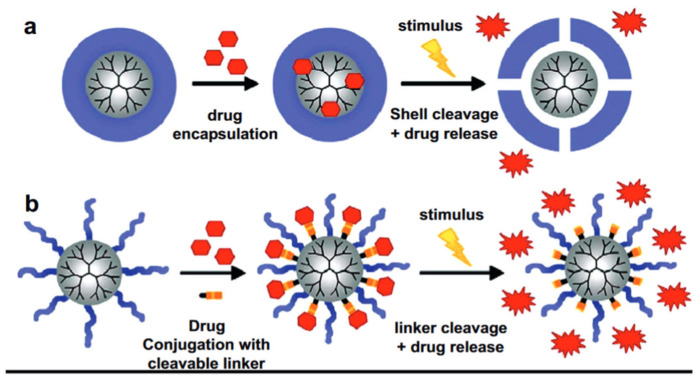
Two main mechanisms for encapsulation and corresponding pH-triggered release mechanisms: (**a**) core–shell structures comprising cleavable shell and (**b**) dendritic scaffolds modified with solubilizing groups, using cleavable linkers for the drug conjugation. Reprinted with permission from [75]. Copyright 2012, Elsevier.

Basically, any class of nanomaterials, both organic and inorganic, can be modified with a pH-responsive release mechanism. Organic materials for pH-responsive targeted delivery systems include polymeric nanoparticles (such as polymersomes, dendrimers, nanospheres, hydrogels, and polymeric micelles); and lipid nanoparticles, including liposomes, solid lipid nanoparticles (SLNs), and nanoemulsions. Organic nanoparticles are well suited for drug delivery because they are biodegradable, water-soluble, biocompatible, and biomimetic. Their surfaces can be easily modified for additional targeting, allowing them to deliver drugs, proteins, and genetic material directly into the tumor cell. Moreover, polymers can be utilized both as a self-sustained pH-responsive particle for drug delivery or as a “gatekeeper” on top of the DDS’s core. Polymeric particles of nano- and submicron sizes with core–shell structures are mostly represented by amphiphilic polymers such as di-block copolymers, di-triblock copolymers, star copolymers, and graft copolymers. The physical properties of such polymeric carriers can be designed by tuning the ratio of hydrophilic and hydrophobic components of the individual block copolymers [73,74]. Polymeric particles ensure drug loading via two principles, namely conjugation of a drug to the monomeric polymer chains and encapsulation (Figure 2).

**Figure 3:**
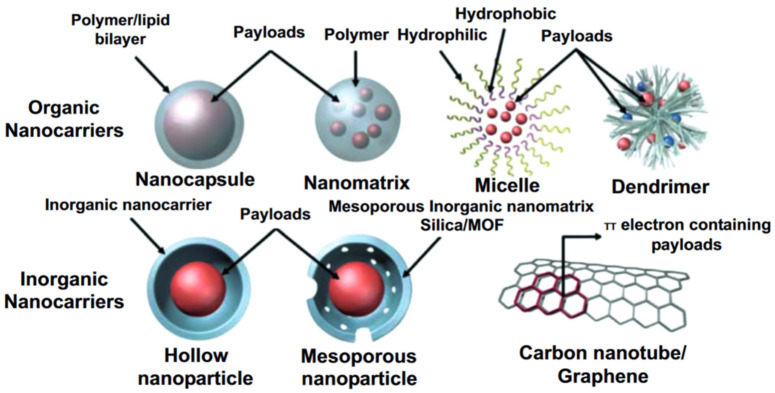
Schematic image of different organic and inorganic drug carriers. Reprinted with permission from [79]. Copyright 2014, American Chemical Society.

Despite all proc of organic nanomaterials, an increased risk of aggregation leading to enhanced toxicity can be mentioned as a main con of organic nanoparticles [76,77]. Inorganic nanoparticles for pH-responsive targeted drug delivery include quantum dots, gold nanoparticles, silica nanoparticles, and magnetic nanoparticles. Most inorganic particles have good biocompatibility and stability and fill those application niches that require properties that are unattainable for organic materials. Limitations on the usage of some types of inorganic particles may be due to low solubility and toxicity, especially when heavy metals are included in their composition. In this regard, a wide range of papers consider hybrid carriers such as metal–organic frameworks (MOFs), which combine properties of organic and inorganic materials. According to Ref. [78], 45.8% of published-by-now articles consider polymers to be a material for the fabrication of pH-responsive targeted delivery systems, 10% consider lipids, 12.5% consider mesoporous silica, and 6.7% consider metals (Figure 3). However, current challenges push researchers to create hybrid carriers that combine properties of different materials; thus, the DDSs developed over the last 5 years are mostly multicomponent capsules or particles comprising inorganic compounds and specific polymers which endow them with multifunctionality.

In Section 3, different types of metal-comprising drug carriers are considered in detail to reveal the beneficial features, such as drug-loading capacity, drug release rates, functionality, etc.

### 2.2. Intratumoral Delivery Strategy

It is assumed that the EPR effect promotes the entrapment of drug carriers in the vessels surrounding the tumor due to their disturbed structure, followed by penetration into the tumor parenchyma. The acidic environment of the tumor “switches” the carrier properties, leading to the cargo release. The pH of extracellular fluid (pHe) in healthy tissues is tightly regulated between 7.35 and 7.45 in order to sustain normal physiology and cellular metabolism. Thus, a normal physiological pHe is a strict constant, while a tumor’s pHe is more acidic, which was independently proved by numerous research groups. Reduced pHe values in tumor is a complex effect which is caused by a number of reasons, including poor blood supply, leading to chronic hypoxia and high levels of acidic metabolic products due to the metabolization of glucose into lactic acid instead of CO_2_ (the so-called Warburg Effect) [80]. The probable reason for this process is the increased production of the enzyme carbonic anhydrase IX, which catalyzes the reversible interconversion of CO_2_ into HCO_3_^−^ and H^+^. It should be noted that carbonic anhydrase IX overexpression is more intensive in the core sites of a tumor producing the internal pH of the cells (pHi) at the core less acidic but making the peripheral pHe of the tumor more acidic [81,82]. Numerical modeling of the data based on spheroid studies revealed that carbonic anhydrase IX maintains a sharp outward-directed CO_2_ gradient, accelerating the CO_2_ excretion, and acidification of the pHe, as well as increasing the pHi. These factors lead not only to an acidic pH in the tumor but also make the acidic environment a condition for the progression of the tumor [59].

**Figure 4:**
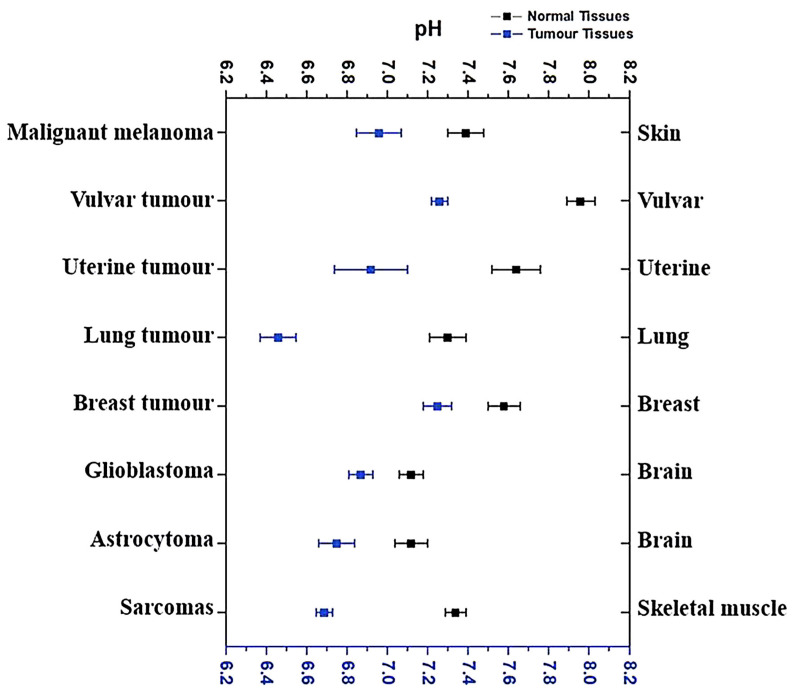
Extracellular pH values of tumor tissues in comparison to pH in the corresponding healthy tissues. Reprinted with permission from [83]. Copyright 2018, Royal Society of Chemistry.

The pH values in different tumor types range between 6.3 and 7.0, which reflect the dysregulation of the acid–base homeostatic mechanisms taking place within solid tumors. Numerous data from the literature comparing the pHe of the tumor tissues and the corresponding normal ones were summarized by G. Hao et al. and represented in Figure 4. Selected results were received with pH-sensitive electrodes as the most common method for intratumoral pH measurements [83]. As shown in Figure 4, the tumor’s pHe is only 0.3–0.7 units lower than that of the corresponding normal tissues. For example, the average pHe of uterine tumor tissues is around 6.92, while the average pHe in a normal uterus is 7.64 [84]. Similarly, the average pHe in malignant melanoma tissues is 6.96, which is only 0.43 lower than that in normal skin tissues (7.39) [85]. Vulvar tumors have an average pHe of 7.26, while the average pHe in normal vulvar tissues is 7.96 [84]. Similar pH differences have also been observed in other tissues, such as brain [86] and lung [87], breast [88], and skeletal muscle [89]. There are only a few types of cancer that have exhibited lower extracellular pH values, in particular, astrocytomas and squamous cell carcinoma with pH values less than 6.0.

Thus, the average gap between healthy tissues and the acidic extracellular environment in tumors is 0.3–0.7. This fact presents a challenging task for chemists since switching a carrier’s state on such a short pH difference is a difficult issue. An analysis of the literature showed that the absolute majority of authors demonstrated pH-responsiveness of DDSs toward pH values in the range of 5 to 6, with an average value of 5.7. However, as was described above, these values of pHe are hardly reachable in the extracellular space of real tumors.

**Figure 5:**
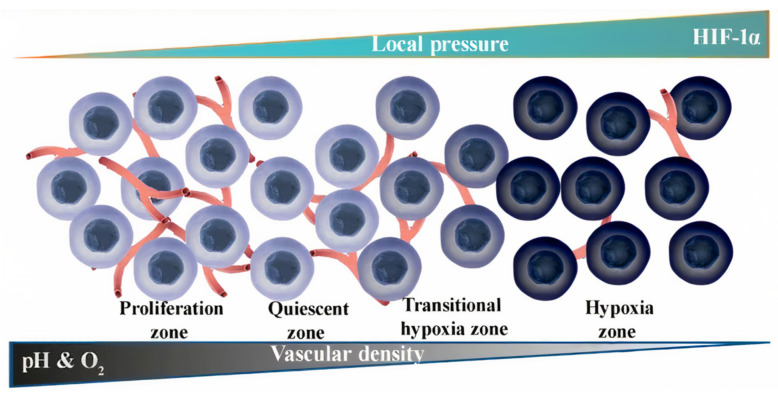
Illustration of tumor tissues. Reprinted with permission from [91]. Copyright 2021, Springer Nature.

Moreover, these values are highly variable, and in general, larger tumors tend to be more acidic, mostly at the late stages of the cancer progression [90]. This makes pH-responsive DDSs hardly applicable at early stages of cancer progression or at small metastatic tumors, which are crucial issues for successful cancer therapy. Moreover, the pH values are not homogenous over the tumor and gradually change from neutral at the periphery to acidic at the central hypoxic zone, which is mainly caused by afferent decreases in tumor vascularization (Figure 5) [91]. This fact significantly mitigates the probability of the pH-responsive DDS administration with the blood flow to the poorly vascularized central zone characterized by the lowest pH level, which also points to the demand for drug-release triggering at a pH slightly lower than the normal one.

### 2.3. Intracellular Delivery Strategy

**Figure 6:**
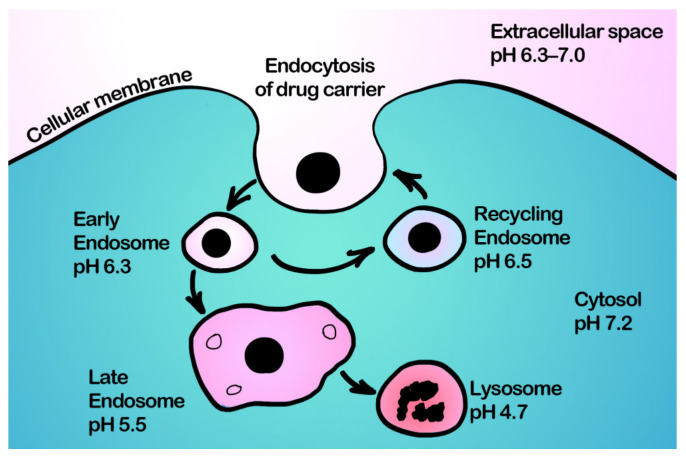
Illustration of pH at different stages of endocytosis.

Intracellular drug delivery via DDSs plays an equally important role in successful cancer therapy, especially for polar molecules poorly permeable through membranes whose therapeutic targets are localized inside the cell. Moreover, this strategy is more promising in terms of pH-responsive drug delivery, since drug carriers are mainly internalized by cells through endocytosis [92], and endosomes are characterized by low pH values (4.5–6.0) [93]. The cytosolic pH of cancer cells in the opposite is close to neutral. Thus, overexpression of carbonic anhydrase IX not only leads to acidification of pHe but also induces a slight shift of pHi to a rather neutral or slightly alkaline region. In normal cells, pHi negligibly differs from pathological ones and hovers around 7.2. Therefore, cancer cells have a higher pHi (pHi > 7.4) than normal (pHi~7.2), which, in combination with acidic tumor pHe, leads to a reversed pH gradient across the cancer cell membrane [83,94]. Nevertheless, the pH within endosomes of cancer cells is in the range of 4.5–6, which is more suitable for inducing pH-responsive release. Thus, during the internalization process, carriers firstly are ingested by a cell with the formation of an early endosome (pH of about 6.3), which then passes into a late endosome (pH of about 5.5) and finally fuses with lysosomes (pH below 5). In its turn, it results in the degradation of the trapped DDS by the action of enzymes. This process is a natural defense mechanism of a cell against extraneous substances (Figure 6).

A large number of researchers suggest releasing therapeutic cargo directly into the endosomes of cancer cells in response to a low pH as an effective method of therapy [95,96,97,98]. However, the penetration mechanism of the drug through the endosome membrane, as well as its stability to the action of enzymes, should be carefully considered. Such a strategy can be applied mostly to non-polar low-molecular-weight chemotherapeutic and immunotherapeutic agents, while the delivery of high-molecular-weight compounds that are sensitive to the action of enzymes and unable to pass through the membrane requires the so-called endosomal escape, which is a specific and complex task and requires the inclusion of endosome-disrupting agents into a DDS. Otherwise, a large fraction of endocytosed therapeutic agents become trafficked to the degradative lysosomal compartment, with subsequent damage to the encapsulated cargo [99,100]. In this regard, the endosomal escape process is preferable to be implemented before the endosomal degradation of the drug carrier to perform the therapeutic effect of the encapsulated active substance [99,100,101,102,103,104]. Currently, endosomal escape is one of the strongest barriers that limits the application of DDSs carrying biological therapeutic agents (such as DNA, RNA, and proteins) to intracellular targets. Despite the increased attention to natural objects capable to endosomal escape, such as viruses and pathogenic bacteria [105,106], it is still difficult for synthetic systems to deliver macromolecules into cytosol and different compartments of a cell [107,108]. Currently most drug carriers employ cationic materials that passively provide swelling and subsequent rupture of the endosome membrane with low efficiency [109,110].

Moreover, the problem of internalization of carriers by cancer cells in tumors is acute along with the following endosomal escape process. Multiple data from the literature show that only a small percentage (0.7–0.9%) of the systemically administered carriers reach the tumor, passing through the EPR effect into tumor parenchyma [47,111], and less than 0.0014% of administered drug carriers are internalized by the cells [112]. Moreover, the features necessary for the effective uptake of drug carriers by tumor cells impede features providing long-time circulation. Positively charged particles more easily interact with cells and become endocytosed because the cellular membrane is negatively charged, but on the other hand, it leads to faster cleaning of the particles by the reticuloendothelial system after administration [113]. To resolve this dilemma, drug carriers capable of changing their charge have been developed. Such carriers are negatively charged in blood circulation, but the acidic microenvironment in tumors reverses particles to a positive charge, which enables enhanced cellular uptake [72]. This effect is obtained by the application of biomaterials, which induce conformational changes in these carriers through various mechanisms, such as protonation, charge reversal, or cleavage of a chemical bond, leading to enhanced interaction of carriers with the cell and promoting cell uptake [43].

### 2.4. Peculiarities of DDS Administration

It is accepted that the passively targeted selective accumulation of nano- and microparticles in tumors occurs due to the EPR effect [114], which is the key process in many cancer research and clinical trials. However, clinical trials have shown poor results in the survival of cancer patients [115], thus pushing researchers to look for alternatives to EPR-mediated delivery of chemotherapeutic compounds [49].

The current data on long-term targeted drug delivery show that it only allows a slight increase in the accumulation of drugs in the target organ or tumor, while most of the drug is distributed throughout the body, accumulating mainly in the macrophage cells of the liver and spleen, leading to a strong toxicological effect and reduced therapy efficiency [111]. These result in a demand for the development of new ways to improve the efficiency and bioavailability of drugs, as well as to reduce off-target toxicity. Multiple data indicate the sequestration of nano- and microsized carriers by the mononuclear phagocytic system as the main reason for their short circulation time in the bloodstream. Thus, a complex approach to improving the therapeutic effect localization includes not only the amelioration of carriers’ targeting but also the implementation of methods reducing the impact on critical organs. Although a few “stealth” systems demonstrated considerable success in stability and prolongation of circulation time, such carriers exhibit poor cellular uptake and slow drug release from endosomes [116,117], thus reducing drug bioavailability and compromising drug efficacy [118,119].

A few approaches, such as protonation and detachment of stealth-agents from particles’ surface, have been suggested to achieve the synergistic benefits of long circulation, enhanced intracellular delivery, and cytoplasmic drug release [75,120,121,122]. These aspects represent the weak sides of the pH-responsive drug delivery concept that should be resolved to provide high efficiency. Thus, the EPR effect allows only a slight increase in the accumulation of particulate drug formulation in the tumor, while the liver still takes the main “strike”, and a vast majority of the systemically administered particles end up in the mononuclear phagocytic system. Moreover, active transcytosis of carriers through the endothelial layer of capillaries ensures their effective delivery and retention in the peripheral interstitial volume but does not provide effective diffusion deeply into the tumor parenchyma [123]. At the same time, not all tumor-supplying vessels are leaky enough to provide traffic of nanoparticles due to their structural heterogeneity [124], resulting in the EPR variety over different cancer types [49,115]. Moreover, a number of studies have shown that the EPR effect is characteristic of rodents, and in humans, it is much less pronounced, as confirmed by clinical studies [49,125,126], which has shown low efficiency in passive targeting of chemotherapeutic agents through the EPR effect. Although the model is consistent, the described approach allows us to reach an efficiency improvement only by fractions of a percent. Thus, about 0.7% of the systemically administered dose of the encapsulated form of the therapeutic agent reaches the target malignant tissue [47].

A significant stride in drug carriers’ efficiency amelioration was also achieved by surface modification of carriers with various gels and polymers. Thus, polyethylene glycol adsorption as the last layer ensures increased circulation time of carriers in the bloodstream and, consequently, improved accumulation in tumor vascular abnormalities [127]. However, the advisability of such surface modification must be carefully estimated, since such a modification results in difficult binding to target tumor cell receptors.

The other innovative approaches in the manner of personalized medicine concept implies the usage of vesicles made from a cytoplasmic membrane, or even entire cells for hiding DDS from the host immune system and better tumor targeting [128,129,130]. A noticeable progress in targeting efficiency was achieved by the carriers’ modification with ligands providing specific reactions with receptors inherent to a particular body site [50,51,131]. This “active” approach has demonstrated efficiency with a wide range of carriers, including liposomes [132,133], micelles [134,135,136], and inorganic nanoparticles [137,138,139,140,141,142]. Compared with passive delivery, it enables increasing the carriers’ accumulation in the area of interest by around 0.9%. According to Ref. [139], this approach not only increases the therapy efficiency via the targeted delivery of drugs but also via overcoming the tumors’ drug resistance. However, it is noteworthy that the application of multifunctional carriers is not a panacea, and the best results can be achieved with an integrated approach that employs both passive and active targeting in combination with methods reducing the effect on healthy body tissues.

However, despite the substantial progress in technology development and the approval of drug carriers by the ministries of health in different countries, they still show very modest survival results in clinical trials [115]. Today, EPR approaches are aimed at increasing the ability of carriers to diffuse into the tumor extracellular matrix. Thus, the decomposition of nanosized carriers into moieties smaller than 10 nm in response to the impact of the tumor microenvironment increases their diffusion into the interstitium and provides better access to target tumor cells; however, this does not fully solve the mentioned problems of the method [143].

The currently existing problems of ERP-based DDSs push researchers to develop new drug delivery approaches, which are less dependent on tumor biology [144]. Recently, an alternative concept of drug delivery based on the Flash drug Release in the Endothelium (FlaRE) of vessels supplying the tumor was proposed [145,146]. This approach implies the accumulation of a DDSs in capillaries of the perivascular leaky regions of the tumor, fast vehicles degradation at neutral pH values providing sharp local bust in active substance concentration, and following drug diffusion according to the concentration gradients across the endothelial wall into the tumor interstitium. For this purpose, carriers with reverse pH responsiveness, capable of releasing cargo at physiological pH (7.4) and “closing” at a lower pH, are supposed to be used. This concept is aimed to eliminate a number of the problems facing researchers in the delivery of various DDSs into tumors, such as the poor outcome of the EPR effect in humans revealed during clinical research [49].

The application of carriers with a controlled release profile does not completely resolve the aforementioned disadvantages of systemically administered encapsulated drugs. Moreover, their application can be a reason for pronounced adverse effects which are not characteristic of conventional formulations. Thus, macrophages, as well as cancer cells, are also characterized by a reduced endosomal pH (pH of about 4 in some cases), which leads to a rapid release of the therapeutic agent from pH-responsive tumor-targeted carriers after their internalization by this type of immune cells. As a result of the sequestration of carriers by the mononuclear phagocytic system, the time of their circulation in the bloodstream is significantly reduced by up to 1 min, which obstructs the process of carriers’ accumulation in the tumor [146]. Moreover, the total percentage of DDSs trapped in the liver in some cases reaches 70% of the dose introduced into the bloodstream, which leads to strong toxicological stress. For example, gold nanoparticles have been shown to remain in liver macrophages for up to 12 months after administration [147]. The process of carriers’ accumulation in the tumor tissue and their sequestration by the mononuclear phagocytic system is determined by a number of parameters, including size, shape, charge, and the nature of the surface [47], and this pushes researchers to search for the optimal carriers configurations for particular tasks.

## 3. Analysis of Metal-Comprising Drug Carriers’ Features

In this section, we consider structures and properties of metal-comprising drug carriers to reveal patterns in their efficiency in terms of drug-loading, release, and cytotoxicity to build the profile of the “ideal” drug carrier in the frame of the described strategies. We employ MOFs and other metal-based carriers as an example since these classes possess a wide variety of compositions and properties, including unique and superior structures with an increasing number of publications over the last 5 years.

### 3.1. Drug-Loading Efficiency of Different pH-Responsive Metal-Comprising DDSs

One of the most important characteristics of DDSs is the amount of an active substance that can be loaded into them, and pH-responsive carriers are not an exception. A high loading capacity is required for the drug vehicle to induce a desired therapeutic effect in the organism. Moreover, it is worth noting that the presence of DDS itself in the body can be the reason for different metabolic issues. Thus, the achievement of the maximal ratio of the loaded drug to the carriers’ mass is preferable to minimize possible adverse effects caused by DDS application. Multiple studies have demonstrated the dependence of this characteristic on intermolecular interactions between drug molecules and carrier materials [148], and DDSs’ structure (surface area, pores size, and internal volume) [149]. Depending on the DDS’s type the carrier–cargo binding can be determined by different kinds of interaction including hydrophobic, electrostatic, covalent, hydrogen bonding, π-π stacking, and van der Waals force [148,150,151,152]. Since these forces depend on the charges of carrier and active substance, to one extent or another, it is important to consider the efficiency of the DDS loading process in terms of their electrostatic complementarity. Surface area, pores’ size, and internal volume are also crucial parameters since they determine the loading capacity of a carrier and the interaction of a carrier with proteins, cells, and tissues. Therefore, the ration between the mass of a carrier and the mass of the loaded drug is one of the most important characteristics of the DDS [148,153].

Usually, the drug-loading capacity (DLC) and the drug entrapment efficiency (DEE) of carriers are used as the main features for the quantitative evaluation of drug-loading efficiency [154,155,156,157]. DLC is the ratio of the weight of an active substance incorporated into the DDS to the weight of the substance-loaded DDS and can be presented as follows:

(1) $$\mathrm{DLC}=\frac{\mathrm{Wt}\;\mathrm{of}\;\mathrm{substance}\;\mathrm{incorporated}\;\mathrm{into}\;\mathrm{DDS}}{\mathrm{Wt}\;\mathrm{of}\;\mathrm{loaded}\;\mathrm{DDS}}\times 100\%$$

where Wt of loaded DDS is the sum of the DDS weight plus the weight of the incorporated substance. In its turn, DEE presents the ratio of the weight of an active ingredient incorporated into the DDS to the total weight of the active substance used for the loading of DDS, and it can be presented as follows:

(2) $$\mathrm{DEE}=\frac{\mathrm{Wt}\;\mathrm{of}\;\mathrm{substance}\;\mathrm{incorporated}\;\mathrm{into}\;\mathrm{DDS}}{\mathrm{Wt}\;\mathrm{of}\;\mathrm{substance}\;\mathrm{used}\;\mathrm{for}\;\mathrm{DDS}\;\mathrm{loading}}\times 100\%$$

Despite both of these metrics being widely used for the evaluation of drug-loading efficiency, DEE is the less suitable metric for comparative analysis of different DDSs, since it depicts the effectiveness of the protocol for drug loading into the carrier, whereas DLC is an indicator of the carrier’s application efficiency as a container for a certain active substance. Therefore, hereinafter, we consider DLC as the main metric for the comparison of different DDS in terms of their loading efficiency. It is also noteworthy that the DLC is not a universal value, and it depends on both active substance and DDS properties. Thus, it is essential to compare the DLC of different carriers for the same substance.

**Figure 7:**
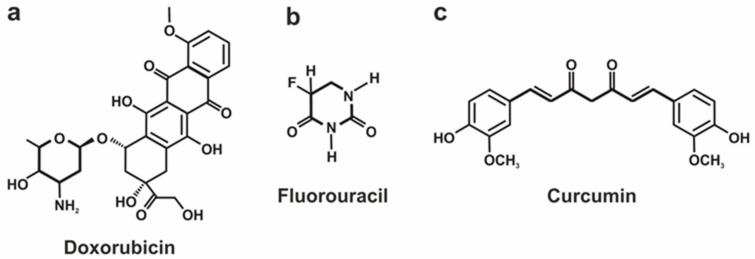
Stereochemical structure of (**a**) Doxorubicin (DOX), (**b**) Fluorouracil (5-Fu), (**c**) and Curcumin (CUR).

For the last five years, an abundant number of articles dedicated to pH-responsive DDS implied for cancer treatment have been published. The list of medications successfully loaded into metal–organic-frameworks- and metal-oxide-based DDSs include more than 10 items listed in Table 1. Based on the data published over the last five years, we can conclude that Doxorubicin (DOX), Fluorouracil (5-Fu), and Curcumin (CUR) (Figure 7) are currently the most frequently used model substances for the evaluation of the pH-responsive DDSs’ efficiency due to their physical, chemical, and pharmaceutical properties. Thus, DOX is a convenient model drug because of its anticancer effect against a broad range of malignant cell lines [158], which simplifies the evaluation of the DDSs’ efficiency and its fluorescent properties [159,160,161], which enable the investigation of the behavior of the drug inside the cell without additional labeling. Moreover, 5-Fu, as well as DOX, is also one of the frequently administered chemotherapeutic agents due to its broad anticancer activity against tumors of the gastrointestinal tract, pancreas, ovary, head, liver, neck, breast, and brain [162]. Moreover, the development of an effective vehicle for 5-Fu delivery is a priority task because of its high systemic toxicity [163], low bioavailability, and short plasma half-life [162]. CUR is considered as a naturally derived polyphenol that possesses a wide range of bioactive properties, including anticancer and anti-inflammatory ones [157]. Moreover, CUR is a coloring agent, which makes it easy to control metabolic issues [164]. In addition, CUR is a hydrophobic substance which makes researchers look for efficient ways to deliver CUR within the body [165,166]. Hereinafter, we will compare the different configurations of drug vehicles in terms of the loading efficiency of the abovementioned drugs.

#### 3.1.1. Doxorubicin Loading Efficiency

DOX is a positively charged molecule at neutral and acidic conditions [167,168] with an average mass of around 543.5 Da and a maximal molecule diameter of around 1.5 nm [169]. The chemical structure of DOX molecules (Figure 7a) enables them to be linked with the carrier surface in both ways covalently [150,151] and through the hydrogen bonding [170,171]. Currently, MOFs are the most popular metal-comprising DDSs applied for pH-responsive DOX administration because of their DLC order of magnitude greater than for MeO NPs (Table 1). A comparative analysis showed that Dextran-modified ZIF-8 [170], UIO-66-NH_2_ with grown Prussian blue (PB) crystals on its surface [171], and hydrothermally reduced NH_2_-MIL-88B(Fe) (Fe-MOF) modified by polyelectrolyte multilayer [172] are characterized by the highest loading efficiency of DOX.

ZIF-8 is nanocrystals consisting of Zn^2+^ and 2-methylimidazole ions that possess a relatively large pores size (3.4–18 Å) [173,174,175], surface area (1244–1630 m^2^/g) [140,170,174,175], and pore volume (0.88 cm^3^/g) [140], resulting in a substantial loading capacity and a positive surface charge ranging from +12 mV to +29 mV [140,174,176,177,178]. As can be seen from Table 1, the DOX loading capacity of ZIF-8-based carriers highly depends on their final configuration and can vary from 10 to 63%. Yongming Chen and co-authors developed the DOX@ZIF-8/Dex configuration comprising ZIF-8 covered with dextran-linked imidazole to improve its colloidal dispersity in aqueous media [170]. The drug loading in this core–shell structure was performed through the hydrogen bonding of DOX with imidazole molecules, and this configuration provided DLC of 63% for DOX, which is the outstanding result among all considered ZIF-8-based vehicles.

UIO-66-NH_2_/PB/DOX is characterized by close to DOX@ZIF-8/Dex DOX loading capacity value (67.4%), despite its more than two-times-lower surface area (570–876 m^2^/g) and pore volume (0.379 cm^3^/g) [171,179]. However, according to Ref. [179], UiO-66-NH_2_ crystals’ pore diameter is around 19 nm, which is 10-fold greater than for the abovementioned ZIF-8-based vehicle. Moreover, the significant DOX loading capacity of UIO-66-NH_2_ can be explained by the carrier’s negative charge (−4.91 mV) [180], which, in combination with the positive charge of the DOX molecule, results in a high DLC. Thus, Jing Wang’s research group found that drug molecules mainly link with the carrier surface via hydrogen bonding between DOX’s hydroxyl group and the carboxyl group of UIO-66-NH_2_/PB [171].

The most outstanding DOX loading capacity (88.4%) has been demonstrated by DDS based on modified NH_2_-MIL-88B(Fe) [172]. The authors explained the high DLC of DDS by its large cavity and specific surface area. However, it is worth noting that Fe-MOF is the needle-shaped nanoparticle with a relatively low surface area (592.2 m^2^/g) if compared with ZIF-8 carriers. The pore size of Fe-MOF is 5.4 nm, which is larger than that of ZIF-8-based DDSs but lower than for UiO-66-NH_2_ ones, which both are inferior compared to Fe-MOF in regard to the DLC. In addition, the ζ-potential of Fe-MOF does not shed light on the mechanism of a high DLC. Thus, the authors noted that the ζ-potential of the empty carrier is +26.9 mV and slightly decreases after its loading with positively charged DOX up to +19.8. In the context of the previously published work [181], which is mentioned as a reference for the method of Fe-MOF synthesis, these positive ζ potential values can be considered a misprint, since MOF progenitors described in Ref. [181] possessed a strongly negative charge (less than −20 mV). Furthermore, the sorption of polymers on the MOF surface after loading with DOX can facilitate a high DLC, thus preventing undesired premature cargo leakage.

**Table 1:** Characteristics of different DDSs utilized for the delivery of considered active substances. drug-loading capacity (DLC), drug entrapment efficiency (DEE), * lipoic acid–curcumin, and ** 10-hydroxycamptothecin (HCPT).

Active Substance	DDS Type	DDS Configuration	Surface Area (m^2^/g)	Pore Volume (cm^3^/g)	Pore Size (nm)	DDS’s ζ Potential (mV)	DLC (%)	DEE (%)	Ref.
Doxorubicin (DOX)	MOF (ZIF-8)	HMS@ZIF	788	0.65	-	-	-	-	[182]
		DOX/HMS	483	0.42	-	-	34	-	
		DOX/HMS@ZIF	1152	-		+31.2	28	-	
		DOX/HMS@ZIF-50	120	-	-	+30.1	44	-	
		BSA/DOX@ZIF	-	-	-	+26.7	10	-	[160]
		DOX@ZIF-8	-	-	-	+27	10	-	[141]
		DOX@ZIF-8@AS1411	-	-	-	−8	-	-	[141]
		ZIF-8	1465.9	-	0.6	+28.9	-	-	[174]
		ZIF-8@DOX	-	-	-	−33.7	43.3	-	
		ZIF-8@DOX@Silica	-	-	-	−32.6	42.7	-	
		ZIF-8@DOX@Organosilica	-	-	-	−34.3	41.2	-	
		ZIF-8	1244	-	1.8	-	-	-	[170]
		DOX@ZIF-8/Dex	1078	-	1.8	-	63	-	
		H-ZIF-8/PDA-CD JNPs	-	-	-	−19.5	-	-	[183]
		HCPT@DOX@H-ZIF-8/PDA-CD JNPs	-	-	-	-	42	-	
	MOF (ZIF-90)	UC@mSiO_2_-RB@ZIF	556.2	0.68	-	-	-	-	[150]
		UC@mSiO_2_-RB@ZIFO_2_-DOX-PEGFA	-	-	-	-	6	-	
	MOF	UCMOFs	-	-	-	+19.1	-	-	[151]
		UCMOFs@Dox@5-Fu	-	-	-	+16.3	16.4	-	
	MOF (UIO-66)	UIO-66-NH_2_	569.595	-	-	-	-	-	[171]
		UIO-66-NH_2_/PB/DOX	-	-	-	-	67.4	-	
		Fe_3_O_4_@UIO-66-NH_2_/Graphdiyne	-	-	-	−23.2	-	-	[167]
		Fe_3_O_4_@UIO-66-NH_2_/Graphdiyne/DOX			-	+5.07	43.8	-	
	MOF (Cu (II)-porphyrin)	Cu(II)-porphyrin/Graphene oxide	352	0.32	4.9	−19.8	-	-	[184]
		Cu(II)-porphyrin/Graphene oxide-DOX	-	-	-	−2.15	45.7	-	
	γ-cyclodextrin-based MOF (CD-MOF)	DOX/γ-CD-MOF	-	-	-	-	-	45	[185]
		DOX/GQDs@γ-CD-MOF	-	-	-	-	-	51.6	
		DOX/AS1411@PEGMA@ GQDs@ γ-CD-MOF	-	-	-	-	-	89.1	
	MOF (NH_2_-MIL-88B)	NH_2_-MIL-88B	-	-	-	+57	-	-	[186]
		DOX@NH_2_-MIL-88B	-	-	-	-	7.4	-	
		DOX@NH_2_-MIL-88B-On-NH_2_-MIL-88B	-	-	-	+86	14.4	-	
	MOF (NH_2_-MIL-88B (Fe))	Fe-MOF	592.2	-	5.4	+26.9	-	-	[172]
		DOX@FeMOF@PSS@ MV-PAH@PSS	-	-	-	−13.5	88.4	-	
	MOF (MIL-101)	MIL-101	4500	-	2.9–3.4	-	-	-	[145]
		MIL-101@DOX	-	-	-	-	36.2 ± 1.4	-	
	MOF	NiCo-PBA@DOX	-	-	-	-	-	19.6	[142]
		NiCo-NiCo-PBA@Tb^3+^@DOX	-	-	-	-	-	16.9	
		NiCo-NiCo-PBA@Tb^3+^@ PEGMA@DOX	-	-	-	-	-	72.2	
		NiCo-PBA@Tb^3+^@ PEGMA@AS1411@DOX	-	-	-	-	-	60.3	
	MOF	Bio-MOFs	935	0.37	3.47	-	-	-	[156]
		DOX/Bio-MOFs	-	-	-	-	39	76	
		CS/BioMOF	438	0.25	3.12	+2.4	-	-	
		DOX/CS/BioMOF	-	-	-	-	48.1	92.5	
	MeO NPs	MnO_2_NPs@Keratin@ DOX	-	-	-	-	8.7	-	[187]
Fluorouracil (5-Fu)	MOF	CS/Zn-MOF@GO	2.22	0.51	35.17	-	-	-	[162]
		5-Fu@CS/Zn-MOF@GO	-	-	-	-	45	-	
	MOF	UCMOFs	-	-	-	+19.1	-	-	[151]
		UCMOFs@Dox@5-Fu	-	-	-	+16.3	24.7	-	
	MOF (UIO-66)	UiO-67-CDC	818.3	0.91	-	+0.229	-	-	[188]
		5-Fu@UiO-67-CDC	-	-	-	-	22.5	-	
		UiO-67-CDC-(CH_3_)_2_	354.3	0.73	-	+22.017		-	
		5-Fu@UiO-67-CDC-(CH_3_)_2_	-	-	-	−0.106	56.5	-	
	MOF	[Zn_3_(BTC)_2_(Me)(H_2_O)_2_](MeOH)_13_	1426	-	0.59	-	-	-	[189]
		5-Fu/[Zn_3_(BTC)_2_(Me) (H_2_O)_2_](MeOH)_13_	-	-	-	-	34.32	-	
Curcumin (CUR)	MOF (ZIF-L)	ZIF-L	-	-	-	+3.8	-	-	[190]
		CUR@ZIF-L	-	-	-	+4.1	-	98.21	
	MeO NPs	N-succinyl-CS-ZnO	-	-	-	−26.1 ± 1.35	-	-	[152]
		CUR-CS-ZnO	-	-	-	−16 ± 1.1	13	69.6	
		ZnO-PBA	-	-	-	−4.7 ± 0.31	-	-	[157]
		ZnO-PBA@CUR	-	-	-	−16.4 ± 0.30	35	27	
		Fe_3_O_4_@Au-GSH	-	-	-	−5	-	-	[191]
		Fe_3_O_4_@Au-LA-CUR/GSH *	-	-	-	−16	-	70	
Camptothecin (CPT)	MOF (MIL)	MIL-101(Fe)-Suc-CPT	1254	0.16	3.6	+6.4	17.6	-	[192]
		MIL-101(Fe)-Click-CPT	143	0.03	3.4	+3.4	18	-	
		MIL-100(Fe)-Suc-CPT	71	0.07	3.5	–27	1.3	-	
		MIL-100(Fe)-Click-CPT	70	0.09	3.6	–45.8	9.2	-	
	MOF	HCPT@DOX@H-ZIF-8/PDA-CD JNPs **	-	-	-	-	9.8	-	[183]
Dihydroartemisinin (DHA)	MOF (ZIF-8)	ZIF-8	-	-	-	+14.9	-	-	[176]
		DHA@ZIF-8	-	-	-	+15.3	14.9	77.2	
		Fe/ZIF-8/DHA	-	-	-	–7.4	42.2 ± 3.3	96.2 ± 3.6	[129]
Quercetin (Q)	MeO NPs	PBA-ZnO	-	-	-	−1.8 ± 0.12	-	-	[39]
		PBA-ZnO-Q	-	-	-	−10.2 ± 0.36	29.83	46.69	
		ZnO-Q	-	-	-	-	17.4	-	[193]
Sonosensitizers Chlorin e6 (Ce6)	MOF	Cu-MOF/Ce6	-	-	-	-	8.7	-	[194]
	MOF (ZIF-8)	ZIF-8	-	-	-	+17	-	-	[178]
		Ce6-DNAzyme@ZIF-8	-	-	-	–22	10	-	
Alpha tocopheryl succinate (α-TOS)	MOF (ZIF-8)	ZIF-8	1485	0.88	-	+22.1	-	-	[140]
		α-TOS@ZIF-8	703	0.25	-	+20.2	43.03	-	
As(III)-drugs	MOF	Zn-MOF-74	1187	-	-	-	-	-	[195]
		As_2_O_3_@Zn-MOF-74	452	-	-	-	11.6	-	
Chloroquine diphosphate (CQ)	MOF (ZIF-8)	ZIF-8				+12.1	-	-	[177]
		CQ@ZIF-8	756	-	-	+9.5	18	-	
Rose Bengal (RB)	MOF (ZIF-90)	UC@mSiO_2_-RB@ZIF	556.2	0.68	-	-	-	-	[150]
		UC@mSiO_2_-RB@ZIFO_2_-DOX-PEGFA	-	-	-	-	5.6	-	
Piperlongumine (PL)	MOF	Fe-TPA	-	-	-	+45 ± 2.8	-	-	[196]
		Tf-Lipo-Fe-TPA@PL	-	-	-	−10.2 ± 0.6	12.3 ± 4.33	78.7 ± 2.98	
Methyl gallate (MG)	MOF (ZIF-L)	MG@ZIF-L	-	-	-	–21	18.05	90.26	[197]
Imatinib	MeO NPs	Fe_3_O_4_@CS/Imatinib	-	-	-	-	52	61	[155]

Therefore, we can assume that the combination of a strongly negative surface charge of the carrier implied for the DOX transportation with a large pore size is the most suitable for DDS. Moreover, we can assume that the sorption of polymer molecules on the MOF surface can prevent the undesirable premature leakage of drugs, thereby increasing its DLC.

#### 3.1.2. Fluorouracil Loading Efficiency

5-Fu is a small (3 × 6 Å) [188] negatively charged [198] molecule with an average mass of around 130 Da (Figure 7b). Similar to DOX, different types of MOF-based DDSs were proposed in the superior majority of studies devoted to the pH-responsive vehicles for targeted 5-Fu delivery (Table 1). Among them, from the viewpoint of 5-Fu loading efficiency, we can emphasize two different configurations of zinc-based [162,189] and one modification of zirconium-based MOFs [188]. Thus, the Zn^II^-based MOF ([Zn_3_(BTC)_2_(Me)(H_2_O)_2_](MeOH)_13_) is nanosized porous spherical crystals comprising Zn^2+^ nudes linked by 1,3,5-benzenetricarboxylic acid (H_3_BTC) and melamine (Me) as organic ligands [189]. This MOF possesses a comparatively large surface area of 1426 cm^2^/g, along with ZIF-8-based DDS and a small pore size of around 5.9 Å. Such a configuration of DDS enables the achievement of a substantial 5-Fu loading capacity (34.32%) (Table 1). Jiying Wang and co-authors, based on the Grand Canonical Monte Carlo simulation, concluded that the 5-Fu molecule links with MOF via hydrogen bond interactions between fluorine and oxygen atoms of 5-Fu and hydrogen atoms of amino and hydroxy groups of MOF.

The other DDS characterized by considerable DLC of 5-Fu is the CS/Zn-MOF@GO hybrid, which is a microspherical porous core–shell structure with a rough surface comprising the core made of Zn-MOF-covered graphene oxide nanosheets and a chitosan shell [162]. The authors reported the extremely small surface area (2.2 cm^2^/g), which is almost three orders of magnitude lower than the surface area of Zn-based MOFs but the equivalent pore volume (0.51 cm^3^/g) which can be explained by the extremely large pore size of CS/Zn-MOF@GO hybrid structures (average pore size of 35.17 nm) (Table 1). Despite the small surface area available for drug binding, the 5-Fu loading capacity of CS/Zn-MOF@GO is also high (around 45%). Authors explain this fact by a number of factors, including drug molecules trapped inside the internal volume of Zn-MOF, hydrogen bonding, π-π stacking, and coordination bond interactions between 5-Fu and Zn-MOF@GO hybrid.

The most promising DDS in terms of 5-Fu loading efficiency is carbazolyl functionalized Zr-based MOF postsynaptically modified via N-quaternization (UiO-67-CDC-(CH_3_)_2_) [188]. The authors considered two configurations of Zr-MOF, namely UiO-67-CDC and UiO-67-CDC-(CH_3_)_2_, and found that the surface area and pore volume of MOF decreased after N-quaternization from 818.3 cm^2^/g to 354.3 cm^2^/g and from 0.91 cm^3^/g to 0.73 cm^3^/g, respectively (Table 1). At the same time, this modification provided a significant rise in ζ-potential of MOF from +0.229 mV to +22.017 mV and its 5-Fu loading capacity from 22.5% to 56.5%. En-Qing Gao and co-authors explain this high DLC by the high affinity between the anionic drug and the cationic MOF and the small size of a drug molecule.

Based on the abovementioned data, we can conclude that ζ-potential is a much more essential characteristic for DDS development for 5-Fu administration than the surface area, pore volume, and size. As we can see, the small size of the drug molecule does not demand the large internal volume from DDS to successfully load it, and the carrier’s surface charge occupies the foreground of this issue.

#### 3.1.3. Curcumin Loading Efficiency

CUR is a polar hydrophobic molecule possessing a commensurable size compared with DOX (Figure 7c) at a remarkably smaller mass (368.4 Da). The negative charge is displaced to the central part of the molecule, while aromatic rings are charged positively [199]. By contrast, DOX and 5-Fu metal-oxide-based nanoparticles are mainly used as carriers for CUR delivery (Table 1). In terms of CUR loading capacity, we can emphasize two configurations of ZnO nanoparticles, namely ones functionalized by N-succinyl chitosan [152] and by phenylboronic acid [157]. M. Reza Khorramizadeh and colleagues proposed to cover ZnO particles with a chitosan layer and then modify its molecules by succinic anhydride to form N-succinyl CS-ZnO particles [152]. CUR molecules in this system covalently bond with N-succinyl CS-ZnO particles via the conjunction of hydroxylic groups of CUR and carboxylic groups of the succinic-modified chitosan molecule. This approach ensures a CUR loading capacity of around 13%. However, the authors mentioned that the particles’ loading with CUR significantly decreased their electronegativity from −26.1 ± 1.35 mV to −16 ± 1.1 mV, resulting in the enhancement of their agglomeration and consequent decrease in the stability in water media.

The modification of ZnO NPs with phenylboronic acid (PBA) provides a two-fold larger drug-loading capacity for CUR [157] than NPs modified with N-succinyl chitosan [152]. Thus, the system proposed by Parames C. Sil’s team demonstrates a DLC of 35%. The PBA adsorption on the surface of amine-functionalized ZnO NPs was provided by their covalent bond, and the CUR loading, in its turn, was ensured by the formation of the chelate ring with ZnO. Moreover, the authors pointed to the superiority of the chelate binding of CUR with the carrier over the covalent one since it provides better sensitivity of DDS toward the changes in the milieu pH.

Since the curcumin molecule includes positively and negatively charged fragments and different functional groups available for bond formation, including ketone, hydroxy, and methoxy ones, currently, the optimal way for curcumin binding with metal comprising DDSs resulting in high DLC has not been established. This, in combination with the promising anticancer and anti-inflammatory properties of curcumin, inspires scientists over the globe to search for new ways to effectively delivery this bioactive molecule via different drug delivery systems.

### 3.2. pH-Responsive Release of the Active Substance from DDSs

The other crucial characteristic of pH-responsive DDSs is the rate of pH-triggered drug release. As discussed previously in Section 2.2 and Section 2.4, currently, there are two opposite concepts to pH-driven drug delivery. The first is the conventional one implying the cargo release at slightly acidic pH level, which is based on the differences in the ipH_i_ and pH_e_ of normal and tumor tissues, respectively [200]. The second one is based on FlaRE and implies the cargo release at pH values close to neutral [145]. Since these concepts place opposite demands on drug delivery systems, they should be considered separately.

#### 3.2.1. Acidic-pH-Triggered Drug Release

The application of the systemically administered DDSs with drug release triggered by acidic pH supposes their long-term circulation in the vasculature system [111,201] and passive [202,203] or active [204,205,206] accumulation in the tumor region with the following drug release at tumor acidic conditions that result in a local raise of a drug concentration up to values providing an effective tumor treatment [207]. At the same time, the release of cargo from the carriers adversely accumulated in healthy tissues should be maximally prolonged and minimized to maintain the drug concentration below a toxic level, provide its gradual clearance, and consequently decrease systemic side effects. Therefore, the “ideal” drug delivery system in the frame of this concept must completely unleash all cargo at an acidic pH and reliably retain it for a long time at the physiological pH level.

As was mentioned previously in Section 2.2, the pHe values in normal and malignant tissues recognizably differ but not drastically. Thus, in healthy tissues, it varies from 7.35 to 7.45, and in cancer ones, from 6.3 to 7.0. Therefore, the difference in the pHe at normal and pathological states is only 0.3–0.7 units. This means that the span between pH values at which the DDS has to retain cargo and release it completely should be as small as possible. Afferently decreasing vascularization of a tumor obstructing DDSs administration via blood flow to the central zone with acidic pHe also points at this requirement.

Mainly, authors demonstrate the sensitivity of proposed DDSs to pH stimuli by comparing cargo release at pH 7.4 and pH 5.0, even though such an acidic pHe is improbable in tumors. Thus, hereinafter, we consider and compare DDSs or the cargo release at pH 5.0 and pH 7.4 to extend the data selection.

**Figure 8:**
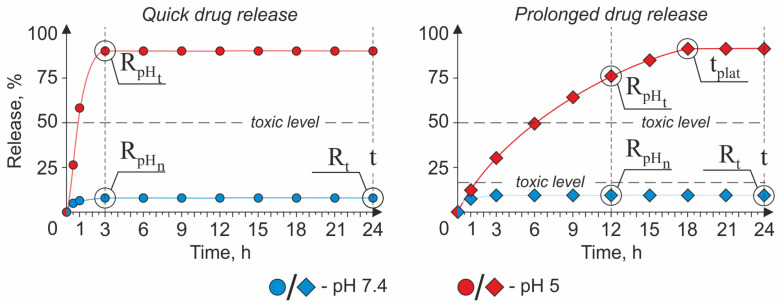
Profiles of the cargo release from DDSs in the frames of quick (**left part**) and prolonged (**right part**) drug release strategies.

To compare DDS efficiency, the formulation of evaluation criteria is necessary. According to mentioned above profile of the “ideal” DDS with low pH-triggered drug release, two evaluation criteria (drug release efficiency and drug retains efficiency) can be formulated. The release efficiency implies how good the DDS is at cargo releasing in a tumor and retaining it in normal tissues. It can be evaluated through the attitude of the amount of released drug at the tumors’ pH to the amount of drug unleashed at normal tissues’ pH after a certain period (Figure 8) and is represented as follows:

(3) $$E_{rel}=\frac{R_{pH_{t}}\;}{R_{pH_{n}}}$$

where $E_{rel}$ is the release efficiency of pH-sensitive DDS (r.u.), $R_{pH_{n}}$ is the cargo release in % at pH 7.4, and $R_{pH_{t}}$ is the cargo release in % at pH 5.0. However, the time period in which the DDS has to completely unleash active substances is still under debate. In some cases, the quick release of cargo during the first hours in an acidic tumor’s conditions is determined as the most suitable strategy, since the sharp increase in the active substance concentration in the target area is required for effective therapy (Figure 8, left part). For example, the application of the DOX-loaded ZIF-90-based system for oxygen-enhanced photodynamic therapy implies the fast release of O_2_ in the tumor microenvironment [150]. Alternatively, a sustainable drug release from DDS (Figure 8, right part) can increase the effectiveness of conventional chemotherapy since the maintenance of the drug therapeutic dose in the tumor area for a long time effectively suppresses cancer cell proliferation and, consequently, tumor growth [91]. However, it is important to emphasize that, in the case of prolonged drug release, its concentration in the tumor area should be maintained above the toxic level to provide a therapeutic effect. Thus, drug delivery systems developed in the frame of these strategies also should be considered separately.

The cargo retains efficiency implies how good the DDS is at retaining active substances in normal tissues for a long period of time. It can be estimated as an attitude of the incubation time of DDS at 7.4 pH to the amount of drug released at such conditions in this time point (Figure 8) and is represented as follows:

(4) $$E_{Ret}=\frac{\;t\;}{R_{t}}$$

where $E_{Ret}$ is the cargo retains efficiency by pH-sensitive DDS at 7.4 pH (r.u.), *t* is the incubation time of DDS at pH 7.4 (24 h), and $R_{t}$ is the cargo release in % in *t* time point.

As was mentioned before, the physical and chemical properties of an active substance and a DDS mainly determine the carrier’s loading capacity. At the same time, the drug release rate also highly depends on both cargo and carrier properties. Therefore, we further compared the drug release rate of different DDSs loaded with the same active substance.

##### The Strategy of a Quick Drug Release

The profile of the cargo release from DDSs created in the frame of the quick drug release strategy is characterized by the averaged curve/pattern shown in Figure 8 (left part). A perfect candidate for a role of a DDS in the frame of this concept should possess high values of the $E_{rel}$ and $E_{Ret}$ criteria. The quick rise (within the first three hours) in active substance concentration in the tumor interstitium allows for achieving the following therapeutic goals: (i) provide a sufficient amount of oxygen for the generation of reactive oxygen species (ROS) in a hypoxic tumor environment during enhanced photodynamic therapy (PDT) [150] and (ii) provide a fast drug concentration boost inside the cancer cells [151,174,186].

MOFs represent a promising class of vehicles based on the abovementioned requirements. In general, MOFs are inorganic–organic hybrid materials composed of metal ions linked by organic ligands [208]. This class of DDS is characterized by high porosity, large porous size, and surface area, resulting in a high payload capacity, biocompatibility and biodegradability, water solubility, and simplicity of the carrier’s functionalization process. Furthermore, several members of this class, including Zeolitic Imidazolate Frameworks (ZIF-n family), Materials of Institute Lavoisier (MIL-n family), University of Oslo (UiO-n family), Dresden University of Technology (DUT-n family), etc., are characterized by pH-responsive drug release that makes them perfect candidates for targeted cancer treatment [209].

Jun Lin and co-authors proposed a ZIF-90-based multilayer pH-responsive composite UC@mSiO_2_-RB@ZIF-O_2_-DOX-PEGFA (URODF) for oxygen-enhanced PDT accompanied by traditional chemotherapy [150]. The URODF has the following core–shell structure: the core comprises NaYF_4_:Yb/Er@NaYbF_4_:Nd@NaGdF_4_ upconversion nanoparticle (UCNP), the first layer of the shell is made from mesoporous silica (mSiO_2_) loaded with Rose Bengal (RB), and the second one is from ZIF-90. Moreover, to improve carriers’ biocompatibility, provide active tumor-targeting, and achieve additional chemotherapy effects, authors modified the ZIF-90 surface with folic-acid-conjugated polyethylene glycol (PEGFA) and DOX via covalent binding. The URODF usage for anticancer treatment implies their intravenous injection, active accumulation in the tumor interstitium, and the irradiation of the tumor region by the near-infrared (NIR) light source with 808 nm wavelength. Particularly, URODF delivered to the tumor’s acidic microenvironment by the circulatory system releases oxygen and DOX due to ZIF-90 shell decomposition providing O_2_ for PDT in oxygen-deprived conditions and boost in chemotherapeutic agent concentration. At the same time, the NIR-excited green emission of the UCNP core induces the RB activation, resulting in the local escalation of the ROS concentration. Thus, the anticancer effect of this composite depends on the release of two different cargos: O_2_ and DOX. The authors have shown that the main part of loaded oxygen is released for the first 90 s of MOFs’ incubation at pH 5.5, which is incomparably faster than the DOX release ratio. In its turn, the DOX release was found to be around 73% after three hours of exposure in acidic conditions and 5% in the neutral one. Based on these facts, we can conclude that the $E_{rel}$ of DOX is 14.6. The $E_{Ret}$ cannot be estimated for URODF, since the maximal time point at which the DOX release was considered is 17.5 h.

The other DDS developed in the frame of this strategy is the dual-drug MOF loaded with DOX and 5-FU proposed by Lining Sun’s group (UCMOFs@D@5) [151]. The UCMOFs@D@5 possesses a core–shell structure, with the core consisting of UCNP (NaYF_4_:Yb/Er@NaGdF_4_) and the shell of ZIF. The mechanisms of drug loading were different for DOX and 5-Fu: DOX molecules were covalently conjugated with the MOF’s surface, whereas 5-Fu molecules were loaded into MOF’s pores via electrostatic adsorption that resulted in differences in a drug release rate. Authors noted a slower drug release for 5-Fu (~48% after 3 h at pH 5.0) compared with DOX (~72% after 3 h at pH 5.0), and this, according to Ref. [210], is caused by the high force of bonding between the base site of 5-FU and the Lewis acid site of metal. The release of 5-Fu and DOX after the same incubation period at pH 7.4 was ~4% and ~9%, respectively, which allows us to determine the $E_{rel}$ for 5-Fu as 12 and DOX as 9. The $E_{Ret}$ after 24 h of incubation was 4 for 5-Fu and 2.4 for DOX.

Hai-Liang Zhu and co-workers also contributed to this field of experimental pharmacology through the development of conventional pH-responsive dual pH- and redox-responsible MOFs for targeted DOX delivery [174]. The redox potential of the media was chosen as co-stimuli triggering the drug release to provide targeted intracellular delivery of the drug since the Glutathione (GSH) concentrations in the extracellular and intracellular media are around 2–20 μM and 1–10 mM, respectively, which significantly impact their redox potential providing conditions for triggered drug release. In their article, the authors compared the drug release rate from pH-sensitive DOX-containing ZIF-8 crystals (ZD) and dual-sensitive organosilica-coated ZD (ZDOS) at different pH levels and DDT concentrations. Based on the DOX release data, ZDOS was characterized by sustainable cargo release and should be considered a system for a prolonged drug release strategy; meanwhile, ZD, in contrast, sharply released almost all cargo for the first hours of incubation at a low pH. The DOX release from ZD after 3 h of incubation in acidic conditions was around 87%, while at the neutral one, it was just 14%. However, the prolonged incubation of ZD for 24 h at pH 7.4 revealed the leakage of a significant moiety of the loaded DOX (around 41%), thus indicating the low stability of ZIF-8-based carriers at the physiological pH. Thus, the $E_{rel}$ of ZD is 6.2, and the $E_{Ret}$ is 0.58.

Xianying Cao’s group proposed the other type of particles classified as MOF for the quick responsive pH-triggered drug delivery—MIL-n family [186]. The authors suggested mono- and dual-responsible drug delivery systems consisting of one (DM) and two (DMM) layers of NH_2_-MIL-88B, respectively, for the intracellular DOX delivery. As in the case of ZDOS proposed by Hai-Liang Zhu, the co-stimuli inducing the drug release from DDM is the media’s redox potential. The main goal achieved by Cao’s team through the growth of the second layer of MOF on top of the first one was the reduction of premature cargo leakage from DDS; thus, the DDM should be considered in the frame of the prolonged drug release strategy. Meanwhile, DM’s drug release profile has demonstrated the unleashing of a significant amount (around 85%) of cargo for the first three hours of incubation at a low pH. However, the undesirable leakage of the active substance at a neutral pH was also high (around 41%); therefore, the $E_{rel}$ of DM can be determined as 2. The $E_{Ret}$ cannot be estimated for DM since the drug release was evaluated only during the first 18 h.

DDSs based on metal oxide NPs can also be effectively applied as a drug vehicle characterized by the low pH-triggered release. M. Reza Khorramizadeh’s research group suggested that ZnO NPs functionalized by N-succinyl chitosan to improve the targeting and bioavailability of a such bioactive compound as CUR [152]. The loading of CUR was provided by its covalent binding with CS molecules adsorbed on the ZnO NPs’ surface. According to the presented data, CUR-loaded ZnO NPs demonstrated the rapid release of an active substance for the first hour, followed by slowing down caused by the fast desorption of weakly bound CUR molecules from the surface of the particle. The CUR release after three hours of incubation in acidic conditions (pH 5.2) was around 67%, and at a neutral pH, it was around 28%, which allows us to estimate the $E_{rel}$ to be equal to 2.4.

Based on these data, we can conclude that DDSs based on Zeolitic Imidazolate Frameworks developed in the frame of this concept are characterized by the best combination of $E_{rel}$ and $E_{Ret}$ (Table 2). It is because of the pH instability of the ZIF-based platforms mainly caused by protonated imidazole in the ligand [211]. However, it is worth noting that DDSs possessing promising properties in the frame of this strategy are characterized by low a DLC if compared with other pH-responsive vehicles (Table 1). At the same time, vehicles with a substantial DLC suffer from drug leakage at neutral conditions [174], which do not fit into the concept of the “ideal” drug delivery systems. Therefore, the prioritized issues in the frame of the quick drug release strategy are the increasing DLC of developing systems and the decreasing undesirable premature active substance leakage at a neutral pH.

##### The Strategy of a Prolonged Drug Release

The prolonged drug release strategy implies the retention of a therapeutic dosage of an active substance in the desired area for a long period due to sustainable drug release. It means that the period between the time point when the release curve crosses the drug’s toxicity threshold and the point of maximal drug release should be as long as possible (Figure 8, right part). Thus, for the $E_{Rel}$ evaluation, we considered $R_{pH_{t}}$ and $R_{pH_{e}}$ after 12 h of incubation and also gave consideration to the time point at which the drug release curve had reached the plateau ($t_{plat}$).

##### Doxorubicin Loaded DDSs

From the standpoint of the prolonged drug release strategy, two configurations of ZIF-based systems and one NH_2_-MIL-88B(Fe) stand out among others loaded with DOX considered in this article. One of them is the DOX-loaded ZIF-8 carrier, the surface of which is functionalized by AS1411 aptamer to ensure cancer cell targeting and reduce the off-target toxicity of the drug [141]. The ZIF-based configuration proposed by Xiaogang Qu’s team demonstrated prominent results. Thus, DOX@ZIF-8@AS1411 released more than half (~68.5%) of the loaded DOX after 12 h of incubation at acidic conditions, while the drug leakage at a neutral pH was negligible (~2%), thus enabling the researchers to determine the $E_{rel}$ to be equal to 34.2 (Table 3). The drug release after 24 h of incubation at neutral conditions also was around 2%, which demonstrated the reliability of this configuration for long-term storage of DOX. However, the DLC of DOX@ZIF-8 lying in the base of the proposed DDS was only 10%, which is insufficient if compared with other ZIF-based configurations (Table 1). The $t_{plat}$ of drug release was reached approximately after 20 h at pH 5, which, despite the low loading efficiency, makes this system a promising candidate in the frame of considering strategy.

Almost the same values of $E_{rel}$ (36.5 a.u.) and $E_{Ret}$ (12 a.u.) pertained to the other MOF-based composition (DOX@FeMOF@PSS@MV-PAH@PSS) described in Ref. [172]. According to the presented data, the NH_2_-MIL-88B(Fe)-based system released around 73% of cargo after 12 h at pH 5 against less than 5% at neutral one. At the same time, this system is characterized by a much higher DLC (around 88.4%) and a more distant $t_{plat}$ time point (24 h) compared with DOX@ZIF-8@AS1411 [141], thus making it more suitable for sustainable DOX delivery. A detailed consideration of this configuration and the mechanisms of DOX loading is presented in Section 3.1.1.

The highest value of $E_{rel}$ criteria was described for the mesoporous silica@zeolitic imidazolate framework (HMS@ZIF) [182]. Rui Cao and co-authors proposed the core–shell-like structure in which the particle of hollow mesoporous silica (HMS) serves as a core loaded with DOX, and the shell is formed by sorption of ZIF nanoparticles on the HMS surface. Due to the high porosity, (788 m^2^/g surface area and 0.65 cm^3^/g pore volume), HMS particles ensure a sufficient DOX loading capacity (34%), and authors suppose the recrystallization of DOX inside the HMS’s cavities to be the result of an ultra-high drug concentration. The pH-responsiveness of DDS was provided via the sorption of the shell consisting of ZIF playing the role of the sealing agent. The authors noted the enlargement of the DDS surface area up to 1152 m^2^/g after the shell sorption, explained by the porosity of the ZIF-shell itself, but the final DLC of DOX/HMS@ZIF decreased to 28% because of the augment of DDS’ mass. The proposed configuration allowed Rui Cao’s group to achieve high reliability in low pH-triggered drug delivery. Thus, around 79% of the drug was released after 12 h at pH 5, while less than 3% at pH 7.4. This $E_{rel}$ can be estimated as 52.6%, which is an outstanding result for the considered pH-sensitive DDS loaded with DOX (Table 3). However, the relatively fast release of cargo during the first 10 h in acidic conditions is a weakness from the standpoint of sustainable drug delivery.

The $E_{rel}$ and $E_{Ret}$ of carriers implicit for prolonged drug release mainly decrease at the enlargement of DLC, as in the case of DDSs considered in the frame of the quick drug release strategy (Table 3) [170,171,184]. It is probably caused by the inability of carriers to effectively link the redundant drug molecules resulting in cargo leakage at pH values close to the physiological ones. The exception is the above-described DOX@FeMOF@PSS@MV-PAH@PSS configuration possessing a high DOX-loading capacity and providing prolonged drug release for 24 h at a low pH, accompanied by negligible drug leakage at the neutral one.

##### Fluorouracil Loaded DDSs

All considered configurations of MOF-based DSSs for prolonged 5-Fu delivery are characterized by poor retention and consequently release efficiency (Table 3). Thus, as previously considered in Section 3.1.2, 5-Fu@[Zn_3_(BTC)_2_(Me)(H_2_O)_2_](MeOH)_13_ [189] and 5-Fu@CS/Zn-MOF@GO [162] possess a relatively low $E_{rel}$ equal to 2.8 and 1.8 a.u. and $E_{Ret}$ equal to 1.3 and 1.2 a.u., respectively. The ZIF-8-based carrier proposed by Srinivas Mutalik and co-authors [154] also demonstrates negligible retain and release efficiency compared with DOX-loaded systems considered in the previous subsection. The 5-Fu@ZIF-8@Lf-TC configuration is the core–shell structure comprising the core presented by titanocene-loaded lactoferrin (Lf-TC) NP and a shell consisting of ZIF-8. The authors did not reveal the mechanism of 5-Fu binding with DDS; however, they pointed out that the 5-Fu loading was performed during the step of ZIF-8 shell formation. The comparative analysis of the considering DDSs reveals the decrease in drug release efficiency, representing the attitude of the amount of cargo released at acidic conditions to the amount unleashed at the neutral one, at the increase of the 5-Fu loading capacity. One of the reasons for this is that the cargo’s leakage gain at pH 7.4 probably caused by the weak binding of a substantial amount of drug with DDS’s surface via hydrogen bonding and π-π stacking interaction and the oversaturation of DDS’s structures available for such interaction by molecules of the active substance.

##### Curcumin-Loaded DDSs

Among the DDS loaded with CUR, the ZnO-PBA@CUR [157] configuration previously considered in Section 3.1.3 possesses the most prospective characteristics. The chelate binding of drug molecules with ZnO-PBA NPs provides a significant DLC (~35%), as well as substantial cargo release at pH 5 and its reliable storage at pH 7.4 ($E_{rel\;}$ = 31, $E_{Ret}\;$ = 4.8) (Table 3). The covalent binding of CUR with DDS in case CUR-CS-ZnO [152] does not ensure such a prominent DLC and sustainable low-pH-triggered drug release, and inferiors in efficiency to the previously described platform (Table 3).

#### 3.2.2. Normal pH-Triggered Drug Release

The pH-sensitive DDSs characterized by the release of cargo at the neutral pH have only recently attracted the attention of the scientific community, and currently, just a few articles devoted to this type of carrier have been published. This strategy, as was mentioned previously in Section 2.4, is based on FlaRE. Within this concept, employed carriers must exhibit a fast release of cargo in the physiological pH values (7.4), and high doses should be reached within 3 h, but the better result probably would be reached at a faster release. The particle size in this case is not that crucial since this concept does not imply the EPR effect, and carriers are only supposed to be trapped by defects in vessels around the tumor. Firstly, this concept was proposed by A.V. Zvyagin’s group in Ref. [145]. In their manuscript, the authors demonstrated, in silico, the effectiveness of the proposed approach compared with the prolonged drug release strategy and corroborated these results with in vitro and in vivo studies. As a DDS, the authors suggested MIL-101 (Fe) MOFs (MIL-101 NPs) comprising iron-based building units linked by terephthalic acid derivatives which are characterized by an outstandingly large surface area (4500 m^2^/g) and narrow pore size distribution (29–34 Å; Table 1). This configuration provided the sufficient DLC of a wide range of model substances, including DOX (36.2 ± 1.4%), Rhodamine 123 (42 ± 3%), and their fast release in PBS within the first 30 min (72.1% and 54.7%, respectively).

The other MOF-based DDS demonstrating a similar pH-sensitive drug release pattern is UiO-67-CDC-(CH_3_)_2_, the chemical structure peculiarities of which were previously considered in Section 3.1.2 [188]. Since En-Qing Gao and co-authors published their work one year earlier than the FlaRE approach was proposed, the authors did not highlight the applicability of their DDS within the frame of this strategy; however, they noted the applicability of the developed carrier for a drug release in blood pH conditions. Deferred from MIL-101 NPs, 5-Fu loaded DDS demonstrated a slower drug release rate and reached the release plateau approximately after 6 h of incubation at pH 7.4 (around 80% of loaded cargo). UiO-67-based DDS was found to be stable at highly acidic conditions (pH 3); however, the increase in pH value up to 5 resulted in a drastic increase in drug leakage (~48% of cargo after 6 h of incubation), which is more than 50% of the amount released at neutral conditions and indicates the low stability of DDS. The authors explain this peculiar behavior of DDS within the framework of the natural bond orbit theory (NBO), which leads to a stronger bond between O–H derived from PBS solution and Zr(IV) in comparison to the carboxyl O atom bond, making Zr-MOF decompose under neutral conditions.

## 4. In Vitro Studies of pH-Responsive DDSs

### 4.1. Cytotoxicity of DDSs

Since the application area of pH-responsive DDSs is the targeted tumor treatment, the evaluation of desirable toxicity on tumor cells and off-target toxicity on the normal ones are the most important characteristics of this kind of carriers, which can be estimated in vitro. As in the case of two previously considered parameters, it is crucial to compare the cytotoxicity of different vehicles loaded with the same active substance because of the diverse mechanisms of action of different drugs. Moreover, it is equivalently important to take into account the differences in structure and metabolic activities of different cell cultures [214] and diversity in mechanisms of kits’ action for cell viability assessment [215,216]; thus, it is eligible to compare different DDSs on similar cell models tested via the same kit.

The common in vitro practice to standardize cytotoxicity results is the estimation/calculation of the half-maximal inhibitory concentration (IC_50_), which indicates how much the testing substance is needed to inhibit estimating biological process by half [217]. Herein, we also used IC_50_ values to compare the targeted toxicity of different drug-loaded carriers, and the linear interpolation method was broadly used for IC_50_ calculation. At the same time, since DDS itself can be a reason for different metabolic issues, it is important to consider the desirable cytotoxicity of drug-bearing carriers through a lens of the toxicity of empty DDS. It is worth noting that the best practice for in vitro cytotoxicity studies include the evaluation of target toxicity on the model cancer cell line and off-target toxicity on the normal one. However, because this process is laborious and expensive, usually the toxicity studies are performed on single predominantly cancer cell line (Table 4). According to the International Standard ISO 10993-5:2009, the conventional threshold level of cell viability value for safe substances is 70% [218]. Thus, the “ideal” drug delivery system should fit the following parameters: (i) drug-loaded carriers should induce cancer cell suppression at the administration of the minimal possible amount of drug, and (ii) the equivalent number of empty carriers should not decrease the cell viability by more than 30% compared to the control. Moreover, the DDS efficiency can be illustrated via the ratio of the efficiency of growth suppression of cancer cells by the capsulated form of the drug vs. the free form. For this, here we introduce the coefficient describing the efficiency of cancer cells’ suppression (ECCS), which presents the ratio of cell viability value at the free drug concentration equivalent to the IC_50_ of the capsulated form to viability at the IC_50_ of the capsulated form (Table 4).

As can be seen from Table 4, there is a significant number of different combinations of the model cell lines and tests assessing their viability. Thus, currently, the high-throughput screening method utilizing tetrazolium-based dyes is the good standard for drug/DDS cytotoxicity evaluation and applies to the majority of studies. MTT (3-(4,5-dimethylthiazol-2-yl)-2,5-diphenyltetrazolium bromide), WST-8 (2-(2-methoxy-4-nitrophenyl)-3-(4-nitrophenyl)-5-(2,4-disulfophenyl)-2Htetrazolium and monosodium salt), and Cell Counting Kit-8 (CCK-8), comprising WST-8, are the most widely used kits for this purpose (Table 4). Despite the fact that both MTT and WST-8 are tetrazolium salts and their principle of action is based on the same basis [220], the comparative analysis of data obtained via these kits is not eligible, since these dyes possess different water solubility that significantly affects the results of cell viability evaluation [221,222,223].

The application of both dyes, as well as many other tetrazolium- and resazurin-based reagents, implies their incubation with cells accompanied by dyes conversion into colored or fluorescent products under the action of viable cells’ enzymes with the following detection, using the plate reader. Dead cells, in their turn, are unable to perform such a transformation, which ensures a reliable method for cell metabolic activity evaluation and, based on it, an indirect cell viability assessment [224]. In the case of tetrazolium salts’ application, the formazans are detectable products characterized by pronounced absorbance at 570 nm for MTT and 460 nm for WST-8. However, it is worth noting that formazan products of MTT tetrazolium are insoluble in water and require solubilization before measurement, while products of metabolism of WST-8 are well soluble in growth media that substantially increase test sensitivity and reliability [221,222,223]. Thus, the conclusions derived from the comparison of data from cytotoxicity tests on the same cell model obtained by different test systems can be imprecise or even wrong.

The other obstacle that mitigates the volume of data that is eligible for comparative analysis is the different periods of co-incubation of DDS with cell lines. As we can see from Table 4, the efficiency of the metabolic activity suppression in cancer cells by drug-loaded DDS is predominantly estimated after 24 h of their co-incubation. However, in some cases, the metabolic issues are assessed after 48 h [39,152,157,191] and 72 h [162,193] of incubation as for the majority of metal oxide-based DDS mentioned in this review, consequently truncating the range of DDS eligible for the comparative analysis.

Next, to evaluate and compare the efficiencies of different DDSs, the IC_50_ is considered to be a function of the DLC and drug release rate. In this regard, systems that were investigated using comparable cell lines, viability tests, and drugs were further analyzed. Here, we chose three groups of papers that investigate the efficiency of DOX-bearing MOF-based DDSs by means of the evaluation of their suppressive effect on MCF-7, HeLa, and 4T1 cell lines utilizing MTT assay.

#### 4.1.1. DOX-MTT-MCF-7

Different MOF-based systems functionalized with both organic and inorganic components were analyzed in terms of their drug delivery properties and cytotoxicity. A meta-analysis of the data clearly showed that a higher release rate provides higher cytotoxic efficiency. Jia et al. developed DOX/HMS@ZIF submicron particles characterized by DLC around 28% capable of releasing ~90% of encapsulated DOX within 10 h that resulted in a superior IC_50_ of 0.12 µm/mL after 24 h of co-incubation of carriers with cells [182]. The ECCS criteria for this configuration was 1.64 a.u. Similarly, Z. Liang reported hybrid BSA/DOX@ZIF particles with DLC around 10% and a high release rate (~80% for 10 h) that also resulted in a great IC_50_ of 0.04 µm/mL after the same time period and substantial ECCS (1.48 a.u.) [160]. Despite the higher DLC and release rate of DOX/HMS@ZIF, if compared with BSA/DOX@ZIF, the superior IC_50_ value pertains to the second configuration. The probable reason is the smaller size of BSA/DOX@ZIF particles promoting cytotoxicity of the system in comparison to larger DOX/HMS@ZIF particles (~100 nm versus 600 nm), as this significantly affects the internalization process and, consequently, intracellular drug delivery. It is also noteworthy that the ECCS of BSA/DOX@ZIF is smaller than for DOX/HMS@ZIF, despite the opposite tendency for their IC_50_ values, indicating the difference in susceptibility of used cell cultures to the action of investigated active substance. A few papers showed hybrid MOF particles with modest release rates (around 65% within 24 h) that resulted in increased IC_50_ ~2.5 µm/mL [156] and IC_50_ ~4 µm/mL [184]. Their ECCS values also were smaller than for previously described ZIF-based carriers: 1.3 a.u for DOX/CS/BioMOF [156] and 1.38 a.u. for Cu(II)-porphyrin/graphene oxide-DOX [184] configurations. A comparative analysis of some carriers was obstructed by the absence of comprehensive data regarding the cytotoxicity of the free drug, which precludes ECCS evaluation [172,185]. We also found that a slow-release rate results in poor cytotoxicity of the system to cancer cell lines. Q. Jia proposed hybrid γ-cyclodextrin-based MOF carriers, which exhibit slow rates of release (maximum of ~80% for 60 h) and corresponding IC_50_ values in the range of 15.76–90 µm/mL in dependence on the composition [185].

#### 4.1.2. DOX-MTT-HeLa

The corresponding tendency was also revealed for the HeLa cell line. Thus, Ren et al. proposed MOF coated by biodegradable organosilica to exhibit pH responsiveness and an excellent release rate of ~65% for 10 h, which ensured high on-target cytotoxicity of the system and IC_50_ of 0.34 µm/mL after 24 h of co-incubation [174]. However, the ECCS for this carrier configuration was relatively low (1.18 a.u.), and this can be caused by the enhanced susceptibility of the used cell culture to drug action determined by its physiological state. Another study showed the DOX@ZIF-8@AS1411 configuration to exhibit a slower release (50% for 24 h) and a corresponding modest cytotoxicity of the drug-loaded carrier with IC_50_ values of 1.64–3 µm/mL [141]. At the same time, this configuration demonstrated prominent ECCS (around 1.8 a.u.), thus indicating its effectiveness if compared with the free form of the drug. It is also noteworthy that the modification of the ZIF-8-based carrier with AS1411 aptamer results in the improvement of IC_50_ (decrease from 3 to 1.64 µm/mL), as well as ECCS (increase from 1.74 to 1.82 a.u.). A newly developed graphene oxide/Cu (II)-porphyrin MOF nanocomposite also showed a modest release rate (~45–65% for 24 h) with a correspondingly low cytotoxicity toward cancer cells (IC_50_ of 14 µm/mL) and ECCS (1.16 a.u.) [184]. A perfect release rate was shown by Xie et al., using UC@mSiO_2_-RB@ZIF-O_2_-DOX-PEGFA nanoparticles; however, a poor DLC of 6% resulted in the low cytotoxic effect of DOX-loaded carriers and large IC_50_ values in the range of 15.5–100 µm/mL in dependence on treatment conditions (presence/absence of laser irradiation) and configuration [150]. Nevertheless, the application of oxygen-enhanced PDT resulted in the elevation of ECCS from 1.6 to 2 a.u. compared to the conventional PDT. Moreover, it indicates the outstanding efficiency of the multifunctional DDS comprising agents for traditional chemotherapy and PDT compared with the conventional drug formulation. The tendency to modest suppression of cancer cell growth at prolonged drug release was also revealed for Fe_3_O_4_@UIO-66-NH_2_/graphdiyne configuration proposed by Xue et al. These particles were shown to provide prolonged release over 36 h (48%) that resulted in reduced cytotoxicity and increased IC_50_ of 20 µm/mL [167]. At the same time, this configuration is characterized by poor ECCS (0.62 a.u.), which points to worse efficiency of capsulated form compared with free drug.

#### 4.1.3. DOX-MTT-4T1

Meta-analysis of drug carriers investigated using the 4T1 cell line has demonstrated a similar pattern. Jia et al. have shown the dependence of DOX release from NiCo-PBA@DOX particles on additional carrier modification with Tb^3+^ and poly(ethylene glycol)methacrylate, and the corresponding improvement of the on-target cytotoxicity and decrease in IC_50_ value at an increase in release rate from 140 µm/mL when <20% of DOX released for 24 h to ~6 µm/mL when 44% of DOX released for 10 h [142]. The ECCS criteria were around 1.5 a.u. for all considered configurations. Nanoparticles of AuNCs@MOF-DOX structure were found to possess high release rates of 67% for 15 h; however, the on-target cytotoxicity was relatively low. Thus, IC_50_ values varied from 6 to 9.25 µm/mL depending on treatment conditions (presence/absence of laser irradiation). The modest suppression efficiency was probably caused by the low DLC of the particles [212]. However, attention should be paid to the substantial increase in ECCS from 0.9 (the application of the particulate formulation) to 1.29 a.u. (combined chemo- and photodynamic therapy). A low cytotoxic effect (IC_50_ = 29.6–100 µm/mL) has also been shown for UC@mSiO_2_-RB@ZIF-O_2_-DOX-PEGFA carriers on 4T1 cell line because of low DLC (6%) despite a high drug release rate of these particles [150].

These data serve as evidence that the drug-loading capacity and release rate of the carriers are crucial parameters for DDS. Studies made using different cell lines demonstrate that prolonged drug release is not preferable under in vitro conditions, and the higher release rates, as well as higher DLC, demonstrate increased cytotoxicity. It not only means the total dose of the drug plays a role but the concentration that can be reached within a cell. In other words, the success in the suppression of cancer cell proliferation directly depends on the active substance concentration inside of them, therefore a promising candidate for intratumoral drug delivery should be able not only to reach a tumor interstitium but also maintain the required therapeutic dose in the region of interest. Obviously, the characterization of the DDS efficiency in terms of drug delivery is an extremely complex process and it is not limited by IC_50_ values in vitro, however, these relations should be taken into account. The introduction and evaluation of ECCS criteria for different configurations of carriers revealed the new aspect important for the evaluation of DDSs efficiency. Thus, we showed the importance of the complex evaluation of IC_50_ and ECCS, since outstanding IC_50_ does not indicate the supremacy of carrier configuration compared to others and can be caused by increased susceptibility of cell culture used in a series of experiments. In its turn, ECCS provides the evaluation of particulate formulation efficiency compared with the free form of the drug in the frame of one study. It is equally important to analyze carriers’ features in terms of internalization ability, together with the ability to circulate in the blood flow without fast infiltration. Another important task is to evaluate the gap between pH values at which the DDS retains cargo and releases it completely to reveal possible outcomes. Moreover, it should be kept in mind that, despite the fact that the long-term release does not demonstrate the high effectiveness of cancer cell suppression in vitro, it does not necessarily mean that the same tendency will take place during in vivo studies.

### 4.2. Internalization of pH-Responsive DDSs

The internalization of DDSs by cells via endocytosis is a key step of the drug delivery to the intracellular therapeutic targets because it results in a release of an active substance in endosomes under the action of a range of factors, including a pH below 5.5, as was described previously in Section 2.3. In general, small carriers with a size <200 nm enter the cell through endocytosis with high efficiency, depending on their size, shape, and surface charge [225,226,227]. However, the demand for the long-term circulation of DDSs in the blood flow drives researchers to modify drug carriers with additional components, such as polyethylene glycol (PEG), and provide a negative charge to avoid infiltration, which impedes features necessary for effective internalization [116,117,228]. In this regard, different techniques enabling changes in carriers’ structure and features in response to acidic pH have been developed to achieve synergistic effects of long circulation without obstructed cell uptake. For example, conformational changes, protonation of shell components, and PEG detachment in response to a slightly acidic pH were shown to promote DDSs ingestion by cells [116,229,230,231,232,233]. Protonation supposes the application of ionizable chemical groups as a component of drug carriers. At the physiological pH, these groups are deprotonated/deionized, but an acidic microenvironment induces protonation or charge reversion, resulting in the structural transformation or disassembly of the carriers [75,121]. The current data demonstrate pH-responsive systems based on the protonation mechanism to be able to enhance cellular uptake into cancer cells. Additional applications of specific ligands, antibodies, etc., have been shown to promote the process of carrier ingestion by cells [113,234,235,236,237].

However, after a drug release from DDS inside the endosome, there is one more obstacle on the way toward the intracellular target—the endosome membrane, which is a substantial barrier for large polar molecules. Taking into consideration the aggressive endosomal milieu capable of harming the active substance [238], there is a demand for the equipment of pH-responsive DDSs with a mechanism of endosomal escape [239]. In other words, the amelioration of carrier properties improving its internalization efficiency by cancer cells is not the sole requirement for tumor-targeted DDS, and rather cascaded-responsive configurations that combine multiple functions within the system are demanded [239]. Therefore, from this point of view, the “ideal” DDS should satisfy at least a few requirements, such as the high loading capacity, long-term circulation, diffusion into tumor interstitium, effective internalization, and sufficient drug release accompanied by specific behavior [43,122].

## 5. In Vivo Studies of pH-Responsive DDSs

The evaluation of therapeutic agent efficiency on in vivo models is a pivotal stage on the way to the clinical trial. This section summarizes recently published studies dedicated to the assessment of pH-responsive DDSs’ efficiency in vivo.

In vivo studies serve several purposes, including the assessment of pharmacodynamic, pharmacokinetic, metabolic, and toxicological issues. Although DDSs are designed to reduce the off-target toxic effects of drugs, there is a certain risk of systemic toxicity induced by carriers themselves because of their properties determined by low dimensional characteristics [240]. The toxicity of DDSs depends on their properties, including size, surface area, charge, solubility, etc. Thus, the superior surface area of NPs leads to their enhanced biological reactivity and specifically intensive production of free radicals such as hydroxyl and superoxide anions. These factors push researchers to carefully consider oxidative stress induced by systemically administered NPs, especially metal-comprising ones, which can cause a considerable inflammatory response. Moreover, an important issue is the metabolic fate of materials utilized for DDSs fabrication in the body. The chemical or enzymatic biodegradation of carriers can provide fragments that are suitable for renal clearance. However, while polymers tend to undergo biodegradation to fragments that are suitable for renal clearance in a reasonable period, the issue of inorganic nanoparticle biodegradation is questionable [241,242]. Thus, gold nanoparticles were shown to be retained within macrophages up to 12 months after their administration [147]. Thus, the possibility of complex toxic processes in the organism makes in vitro studies insufficient in terms of the toxicological evaluation of DDSs and makes in vivo methods a basic-routine tool for carriers’ safety characterization.

Utilizing the endogenous pH as a trigger initiating the cargo release by stimulus-responsive carriers has multiple advantages, encompassing extensive applicability and the absence of the demand for an external trigger. At the same time, authors attribute the low accuracy of drug delivery and its release in non-targeted sites of the organism to the disadvantages of a pH trigger, which are mainly caused by shifts in acid–base homeostasis in tissues in response to a wide range of physiological and pathological (inflammatory changes) states [41]. The meta-analysis of data presented over the last 5 years indicates the common tendency to the enhancement of the antitumor effect at the application of pH-responsive drug carriers in various models of malignant neoplasms typified by increased tumor regression [74]. The heterogeneity and abnormal permeability of the vasculature in a tumor are important factors that contribute to the local accumulation of DDSs, which, in combination with a pH-responsive mechanism of cargo release, provide conditions for targeted cancer therapy. However, according to Ref. [46], on average, only 0.7% of injected carriers reach tumors, and this reduces the efficiency and selectivity of drug delivery and points to the importance of nanomaterials properties for successful therapy. Drug carriers for pH-responsive targeted delivery should possess a number of features that ensure their stability in the bloodstream, penetration through histohematological barriers, enhanced EPR effect, and internalization of DDSs by tumor cells [51].

### 5.1. Polymers-Based DDSs

As was described above, different vehicles, ranging from inorganic composites to polymeric particles, demonstrate pH-triggered modification/degradation. Thus, both natural and artificial polymers have been employed as pH-responsive materials for the DDSs’ design. The biomedical application of carriers poses a range of requirements for the employed materials, including biocompatibility, biodegradability, and the capability to encapsulate a wide range of active substances.

Estrone-modified glycol-chitosan pH-responsive NPs (GCNP-ES) were studied in vivo for the targeted delivery of paclitaxel (PTX) in MCF-7 xenograft mouse model. The application of PTX/GCNP-ES demonstrated an increase in accumulation of the active component in the tumor tissues, improving the tumor growth inhibition index up to 81.4%, in comparison to PTX/GCNP (69.4%) and PTX solution (48.8%). Moreover, in vivo studies of PTX/GCNP-ES have not revealed histological or hematological toxicity [243]. An increase in the accumulation of doxorubicin–triphenylphosphine in tumors has been shown in vivo by the application of pH-responsive NPs consisting of a drug-loaded polylactic and glycolic acid core covalently “wrapped” in a crosslinked bovine serum albumin shell [244]. The shell was suggested to minimize interaction with serum proteins impeding target recognition and macrophages ingesting carriers. The shell of the carriers was additionally functionalized with a pH-sensitive ATRAM peptide to promote the internalization of the carriers by cancer cells in the acidic tumor microenvironment. The systemic administration of these particles also resulted in a significant reduction in the 4T1 tumor volume in mice [244]. Another example of successful DOX delivery was demonstrated by Shiyong Song et al., with polymeric microcarriers comprising a pH-sensitive nanogel based on hyaluronic acid. The intravenous injection of the pH-responsive nanogel to animals with H22 tumor xenografts led to a 10-fold decrease in tumor volume on the 15th day of treatment. A histological analysis revealed significant areas of tumor tissue necrosis. At the same time, DOX-loaded nanogels showed no systemic toxicity [245].

To overcome the limitations of reduced EPR effect, polymeric carriers capable of changing their conformation and structure within the body at different stages of the targeted delivery have been developed. Mice models of human pancreas cancer—BxPC-3, which is resistant to cisplatin human lung cancer; A549R; and metastatic breast cancer, 4T1—were employed to demonstrate the efficiency of the iCluster NPs for the targeted delivery of cisplatin [238]. The iCluster system is an NPs bunch that is capable of decomposition into smaller entities (single NPs) as it overcomes biological barriers in the tumor environment. The initial size of iCluster (around 100 nm) favors the stable circulation of carriers in the bloodstream and accumulation in a tumor. In its turn, the tumor’s acidic pH triggers local cleavage of iCluster into much smaller (around 5 nm) dendrimers, which improve carriers’ penetration into tissues and drug influx, sequentially. In vivo studies have shown that free drug administration inhibits BxPC-3 tumor growth by 10%, while the iCluster system inhibits growth by 95% [246].

At the same time, the low stability in the bloodstream and targeting inefficiency of polymeric carriers were largely evidenced in the literature. These disadvantages were suggested to be a result of the interaction of polymeric carriers with serum proteins, as this affects the target recognition ability and leads to nonspecific distribution in the body [247]. Palanikumar et al. showed the application of silica nanoparticles coated with a non-covalent disulfide-bridged polymer to exhibit a higher EPR-effect, accumulation of hydrophobic drug, and therapeutic effect compared to corresponding self-assembled BcP micelles [248].

### 5.2. Liposome-Based DDSs

Targeted delivery systems based on liposomes loaded with anticancer drugs, including DOX, daunorubicin, and vincristine, are currently under clinical trials [249]. Lipid-based carriers have also been modified with pH-responsive components and have been shown to be effective in targeted drug delivery. The permeability of the liposomal membrane alternates in response to a change in pH by means of protonation/deprotonation of functional groups, leading to morphological and structural changes in lipid bilayers and ensuring the release of the drug. In particular, the efficiency of pH-responsive liposomes modified with EphA 10 in the delivery of triphenylphosphine docetaxel was demonstrated in MCF-7 tumor model. Systemic administration of the proposed lipid-based drug formulation induced significant antiproliferative, antiangiogenic, and proapoptotic effects in laboratory animals [250].

The main factor limiting the application of lipid carriers, including pH-responsive liposomes, is their rapid absorption by phagocytes of the reticuloendothelial system, which leads to a very short circulation time. To prolong the residence of liposomes in the circulatory system, pegylated phospholipids (PEG lipids) are embedded into their membranes, and this prevents the phagocytosis of such DDSs, while, at the same time, reducing the stability and pH responsiveness of carriers [251]. A comparison of DOX delivery efficiency by pegylated and non-pegylated pH-responsive liposomes in 4T1 tumor-bearing BALB/c mice revealed that the inclusion of PEG in the carrier’s structure reduces the antitumor activity [252]. Similarly, to reduce the systemic toxicity of docetaxel (DTX) and increase its therapeutic potential, pegylated pH-responsive liposomes functionalized with anti-VEGF antibodies (VEGF-PEG-pH-Lipo-DTX) have been developed. However, the conjugation of antibodies to the PEGylated surface of liposomes reduced their circulation time. VEGF-PEG-pH-Lipo-DTX was eliminated from the bloodstream faster than PEG-pH-Lipo-DTX [253] since antibody disposition on the PEG cover negates its “stealth” effect.

Attempts have been made to increase the affinity of pH-responsive liposomes to the tumor using the TAT (transactivator of transcription) peptide. However, in vivo studies showed that TAT reduces the therapeutic efficacy of the drug due to inefficient accumulation in the tumor and a higher rate of release into the bloodstream [254]. Wang et al. demonstrated successful inhibition of GL261 glioma through the systemic administration of PTX-loaded polymersomes (PLs) to animals in the ventral striatum. The results of PTX@PS treatment showed over 99% inhibition of glioma growth in vivo due to the sustained release of PTX without the occurrence of drug resistance. The authors showed that an acidic microenvironment similar to the tumor induces the release of up to 79.79% of PTX from the synthesized nanosystem within 19 days [255]. André Luis Branco de Barros et al. showed high antitumor efficacy of pH-responsive liposomes (SpHL) loaded with DOX and coated with folic acid to enhance selective accumulation in tumor sites. Intravenous administration of the SpHL-DOX-Fol hybrid liposomes to 4T1 tumor-bearing mice resulted in a 68% inhibition of tumor growth and a sharp decrease in lung metastasis. Moreover, a histological analysis of tumors after SpHL-DOX-Fol treatment showed more extensive areas of necrosis compared to treatment with free DOX or with particles uncoated with folic acid [256]. Jong Oh Kim et al. developed biocompatible pH-responsive liposomes modified with anti-CD25 antibodies and conjugated to T-regulatory cells to enhance stability, sustained circulation, and localized release of interleukin-2(IL), anti-ligand-1 (PD-L1), and imiquimod (IQ), thereby increasing their ability to provide antitumor immunotherapy. Intravenous administration of these carriers to mice with B16/BL6 melanoma resulted in a five-fold decrease in tumor volume, with a high content of apoptosis and necrosis. Notably, the engineered liposomes were specifically transported into the B16/BL6 tumor microenvironment and exhibited long retention times (up to 72 h) [257]. Guangya Xiang et al. showed the antitumor efficacy of pH-responsive liposomes loaded with DOX and imatinibine and targeted to cell folate receptors. After intravenous administration of liposomes to animals with MCF-7/ADR xenograft tumors, effective tumor inhibition was observed with minimal toxicity to healthy organs. Moreover, liposomes provided the excellent long-term circulating ability and sensitivity to the acidic environment of the tumor [258]. Xiuli Zhao et al. reported the antitumor efficacy of pH-responsive liposomes loaded with DOX and small interfering RNA (siRNA). The systemic administration of the liposomes to MCF-7 tumor-bearing animals showed high efficiency of cancer cells suppression (the inhibition rate was 35.8%). An additional coating of liposomes with polyethylene glycol increased the rate of tumor inhibition to 50.13% primarily by increasing the circulation time of liposomes in the bloodstream [259].

### 5.3. DDSs Based on Mesoporous Silica

Mesoporous silica particles should be emphasized as one of the most promising inorganic drug carriers. Mesoporous silica is safe for systemic administration in concentrations up to 40 mg/kg and does not cause acute or chronic toxicity. Moreover, mesoporous silica particles are biodegradable and can be completely eliminated from the body. Interestingly, the bioavailability of mesoporous silica particles does not depend on the route of administration but depends on the particle size. Thus, the enlargement of particle size from ~32 to ~142 nm leads to a monotonic decrease in systemic bioavailability, regardless of the administration route, with a corresponding accumulation in the liver and spleen [260]. In recent years, a system for pH-responsive and visualizable drug delivery based on mesoporous silica particles and sodium alginate doped with gadolinium (Gd) was developed. These particles had a spherical shape with an average size of about 83.2 ± 8.7 nm. The results of the in vivo safety assessment and hemolysis analysis confirmed the high biocompatibility of this system. MRI experiments on a mouse model of breast cancer showed particle accumulation in the 4T1 tumor, which points to the perspective of proposed systems application in pH-responsive drug delivery, and MRI imaging for cancer diagnosis and treatment [261]. Besides mesoporous silica particles, hybrid carriers based on them have also been extensively studied in vivo. Thus, silica particles coated with polyacrylic acid (PAA) and pH-responsive lipid (PSL) have been developed for synergistic delivery and dual pH-responsive sequential release of arsenic trioxide (ATO) and PTX. Additional modification of silica particles with the F56 peptide provided an increase in the specificity of delivery to tumor cells. In vivo testing of these particles in mice with tumors derived from MCF-7 demonstrated that the co-delivery of ATO and PTX using mesoporous silica NPs coated with LP and PSL significantly increased antitumor activity. Additional modification of the particles with F56 ensured increased selectivity accompanied by a better inhibitory effect on tumor growth [262].

### 5.4. DDSs Based on Metal Oxide NPs

Metal nanoparticles were the fourth most frequently used class of carriers among pH-responsive DDSs in the last 5 years [78]. Studies have shown that metal oxide nanoparticles (such as zinc oxide) exhibit high cancer cell selectivity, drug retention, and controlled release. Many types of metal oxide nanoparticles show low toxicity and good biocompatibility. For example, ZnO nanoparticles rapidly degrade to Zn^2+^ ions at a pH below 5.5. However, as we described above, pH values lower than 6.2 are improbable in tumor interstitium, and the only way to reach such an acidic environment is the delivery into endosomes. Nevertheless, zinc oxide nanoparticles exhibit a cytotoxic effect on tumor cells through mitochondrial dysfunction, the release of ROS, lipid peroxidation, and DNA damage. In addition, detection simplicity due to intrinsic fluorescence, simple synthesis methods, and low cost makes zinc oxide particles suitable nanocarriers for cancer treatment.

In the last 5 years, researchers tended to use hybrid nanoparticles combined with organic materials to endow the system with multifunctionality. For example, Parames C. Sil et al. synthesized PBA-conjugated zinc oxide nanoparticles (PBA-ZnO) loaded with quercetin (a bioflavonoid widely found in plants). The presence of PBA fragments on the nanoparticles’ surface facilitated targeted delivery of quercetin to cancer cells. Moreover, quercetin-loaded PBA-ZnO nanoparticles (PBA-ZnO-Q) demonstrated pH-responsive drug release within the tumor. In vivo studies showed systemic administration of PBA-ZnO-Q to induce apoptotic death of MCF-7 breast cancer cells by increasing oxidative stress and damage to mitochondria [39]. The cytotoxic potential of the nanohybrid is explained by authors as a combinatorial cytotoxic effect of quercetin and ZnO on cancer cells. A similar DDS was employed to deliver Curcumin, which has strong anti-inflammatory, anticancer, and anti-angiogenic properties. Intravenous administration of ZnO-PBA-Curcumin in vivo effectively reduced tumor growth in mice with Ehrlich ascitic carcinoma. A decrease in tumor cell multinucleation was observed in response to ZnO-PBA-Curcumin treatment. Moreover, the biosafety studies of hybrid nanoparticles did not reveal signs of hepatotoxicity and renal toxicity. Guang Yang et al. demonstrated keratin to be an efficient platform for the synthesis of metal oxide nanosystems, including manganese dioxide nanoparticles and gadolinium oxide nanoparticles characterized by excellent colloidal stability and biocompatibility. Nanoparticles synthesized using keratin were shown to exhibit a pH-responsive release of DOX, primarily due to the cleavage of disulfide crosslinks between keratin chains at acidic pH. In vivo biodistribution experiments have shown that hybrid nanoparticles accumulate in the liver after 7 days of intravenous administration and are almost completely eliminated from the body 15 days after injection. Moreover, these particles ensure excellent contrast of MRI signals in the tumor in vivo. In addition, the systemic administration of these particles does not induce damage in healthy intact tissues and organs, thus indicating the good biocompatibility of the nanosystem [187]. Active targeting was demonstrated by gold nanoparticles coated with the naturally derived pH-responsive short tripeptide sequence (Lys-Phe-Gly or KFG), designed for DOX delivery. This targeted DDS demonstrated substantial suppression of cancer cell proliferation and BT-474 tumor growth in mice [263]. However, inorganic nanoparticles can be a reason for metabolic issues; for example, gold nanoparticles have been shown to remain in liver macrophages for up to 12 months after administration [147]. Such long-term outcomes should be carefully considered to select the right carrier for a particular task.

### 5.5. MOF-Based DDSs

To date, a wide range of MOF’s modifications have been developed, and some of them demonstrated prominent results during in vivo testing. Zhang et al. investigated the therapeutic efficacy of zeolite imidazolate backbone (ZIF-8) nanocarriers’ design for DOX and acetazolamide (ACE) delivery. The nanocomposites showed excellent antitumor efficacy after a single intratumoral injection against hepatocellular carcinoma in vivo. Under the influence of ultrasound, (DOX + ACE)@ZIF-8 tended to accumulate directly at the tumor site and invade cancer cells through endocytosis pathways. Moreover, (DOX + ACE)@ZIF-8 showed minimal damage to healthy tissues and adverse hematological effects, demonstrating high biocompatibility [96]. Jing Wang et al. reported nanocomposites based on a porous organic framework functionalized with 8-hydroxyquinoline and loaded with 5-Fu (5-FU@COF-HQ). Intratumoral injection of 5-FU@COF-HQ to B16 melanoma mice resulted in the formation of extensive areas of necrosis and apoptosis [264]. The cargo release from 5-FU@COF-HQ at an acidic pH is determined by the presence of a large number of conjugated nitrogen atoms, including quinoline groups and C=N in the organic framework. Moreover, according to Ref. [264], these MOFs demonstrated low toxicity and good biocompatibility in vivo. Tan et al. proposed nanocomposites loaded with DOX and celecoxibine (Cel) (Dox/Cel/MOF@Gel) based on MOF coated with a pH-responsive hydrogel. Intratumoral injection of these nanocomposites demonstrated high efficacy in SCC-9 tumor inhibition in vivo, inducing tumor apoptosis and regulating tumor angiogenesis, due to the synergistic action of DOX and Cel. The efficiency of tumor inhibition after DOX/Cel/MOF@Gel administration was four and seven times higher compared with the administration of free DOX and Cel, respectively. Such treatment has been found to result in a significant reduction in systemic toxicity and no apparent damage to intact organs [265]. However, the abovementioned MOF-based systems imply their intratumoral administration via injections, and this is applicable only for accessible tumors and, consequently, strictly limits the application scope for these formulations.

The other approach to MOFs’ administration implies their intravenous injection, followed by penetration into tumor tissues through the EPR effect. Wenhe Zhu et al. demonstrated the excellent therapeutic efficacy of ZIF-8-based carriers loaded with dihydroartemisin (DHA). The authors showed that the H22 tumor inhibition coefficient in the group of mice treated by intravenous injection of DHA@ZIF-8-NPs was higher (76.7%) than in the group treated by free DHA (49.2%). Moreover, a significant percent of dead cells through the necrotic pathway were found in the tumor after treatment with DHA@ZIF-8-NPs. Jing Wang et al. demonstrated the therapeutic effect of the other hybrid MOFs (UIO-66-NH_2_) modified with PB and loaded with DOX (UIO-66-NH_2_/PB-DOX) on cervical carcinoma model. UIO-66-NH_2_/PB-DOX significantly inhibited tumor growth, which appeared to be associated with the synergistic effects of chemotherapy and chemodynamic therapy [171]. Shuxian Meng et al. showed the antitumor efficacy of a multifunctional three-dimensional DDS (magnet@FUGY/DOX) based on the hybridization of a MOF and graphdiyne (FUGY), which provides efficient drug release at a pH of 5.0. The intravenous administration of Magnet@FUGY/DOX demonstrated a significant antitumor efficacy against HeLa-driven neoplasm with a tumor inhibition ratio of 77.8%. Fluorescence imaging of HeLa-bearing mice has shown that the application of magnet@FUGY/DOX can increase the uptake of the drug by the tumor [167]. Wang et al. reported combined gene and photodynamic therapy of MCF-7 breast cancer, using ZIF-8 modified with deoxyribozymes known as powerful agents for gene therapy, and the photosensitizer chlorin-Ce6. The intravenous administration of nanocomplexes showed their excellent ability to accumulate at the site of the tumor. Simultaneous delivery of functional DNA and Ce6 to the tumor site resulted in deoxyribozymes-mediated inhibition of the transcription factor EGR-1 (early growth response protein 1), along with photogenerated cellular apoptosis [178].

Besides the hybrid nature of the abovementioned carriers, researchers often provide additional surface coating for DDSs to ensure improved functionality, such as enhanced selectivity of their accumulation in the tumor site or improved endocytosis. In this way, ZIF-based NPs loaded with apilimod (Ap) were coated with a solid lipid shell with embedded pH-responsive linkers (L) based on oleylamine (OA) modified with 3-(bromomethyl)-4-methyl-2,5-furandione (MMfu) and polyethylene glycol. The acidic tumor microenvironment induces the detachment of hydrophilic polyethylene glycol and MMfu, leading hydrophobic OA to be “opened”, which, in its turn, increases the uptake of the carriers by cancer cells. The ZIF@SLN#L NPs induce the production of ROS within cancer cells. At the same time, Ap released from Ap-ZIF@SLN#L also promotes the intracellular generation of ROS and lactate dehydrogenase. In vivo experiments on a pancreatic tumor mice model demonstrated a significant reduction of tumor volume and an increase in survival rate at the application of the Ap-ZIF@SLN#L system. At the same time, the authors note no histopathological changes in the main target organs (heart, liver, spleen, lungs, and kidneys), as well as a normal level of serum transaminase activity after the administration of the proposed system, thus indicating the absence of significant toxic effects [266]. Piaoping Yang et al. developed a nanoplatform based on ZIF-8 NPs (defined as α-TOS@ZIF-8) loaded with unstable and hydrophobic D-α-tocopherol succinate (α-TOS). The nanocomposite was coated with a hyaluronic acid shell to form the HA/α-TOS@ZIF-8 nanoplatform. In vivo experiments in mice with cervical tumor (U14) demonstrated tumor reduction on the 14th day of the experiment after intravenous administration of HA/α-TOS@ZIF-8. The study confirmed the tumor-specific release of α-TOS as a result of the destruction of hyaluronic acid at an acidic pH, followed by ZIF-8 degradation and consequent cargo release [140]. Zhang et al. reported the effective inhibition of subcutaneous HeLa xenograft tumor after intravenous injection of a pH-responsive CAMEL-R nanocomplexes design for the targeted delivery of siRNA synthesized on the basis of MOFs coated with the cancer cell membrane derived from HeLa. Animal treatment with CAMEL-R demonstrated 15 times more effective inhibition of tumor volume compared with the control [130].

Promising results have also been achieved using additional functionalization of MOFs with inorganic components. Zhang X. et al. reported hypoxia-responsive copper MOF particles (Cu-MOF NPs), which are copper clusters linked by organic ligands loaded with the sonosensitizer Ce6. According to Ref. [194], Cu-MOF-Ce6 nanoparticles, after their systemic administration to MCF-7 tumor-bearing mice via injection into the caudal artery, effectively accumulate deep in the tumor interstitium due to the EPR effect, where hypoxic conditions trigger carriers’ degradation, followed by the release of Cu^2+^ and Ce6. Internalized Cu^2+^ ions react with tumor histamine, depleting the latter and reducing Cu^2+^ to Cu^+^, which subsequently reacts with endogenous H_2_O_2_ to form cytotoxic hydroxyl radicals (OH). In addition, ultrasonic irradiation (2 W/cm^2^) of tumor interstitium with accumulated Cu-MOF-Ce5 showed the best ability to suppress tumor growth [194]. Zeng L. et al. demonstrated the therapeutic efficacy of DOX encapsulated in ZIF-8 particles with built-in gold solasters (AuNCs@MOF-DOX nanocomposites) via intravenous administration to laboratory mice in vivo. Combined PDT/chemotherapy using the proposed system is caused by the simultaneous release of AuNC and DOX into tumor parenchyma [212]. Hai-Liang Zhu et al. synthesized spherical ZIF-8 particles loaded with DOX and coated by a biodegradable organic silica shell containing many disulfide bonds acting as a “gatekeeper”. The authors demonstrated efficient accumulation of ZIF-8@DOX@organosilica nanocomposites in a HeLa tumor after intravenous administration and outstanding therapeutic efficacy (inhibition rate of 89.4%). Moreover, necrosis and pathological changes were observed in the tumor zone, thus confirming the excellent therapeutic efficacy of the DDS developed by the authors [174]. Miao Du et al. demonstrated the effective therapeutic ability of MOF particles based on γ-cyclodextrin (γ-CD-MOF) containing graphene quantum dots, providing fluorescence properties, and pH-sensitive poly(ethylene glycol) dimethacrylate (PEGMA), promoting a stimulus-responsive controlled release of DOX. Aptamer AS1411 was chosen as a targeting agent for tumor cells. In vivo experiments in MCF-7 tumor-bearing mice demonstrated tumor reduction on the 14th day after intravenous administration accompanied by a few side effects [185]. Quanyan Liu et al. reported biomimetic ZIF-8 NPs doped with iron ions (Fe^2+^) and loaded with DHA. Following the intravenous administration of particles to laboratory animals, the combination of DHA and Fe^2+^ released from ZIF-8 nanoparticles induced a 90.8% reduction in tumor growth in the a HepG2 human hepatocellular carcinoma model. Moreover, the nanocomplex showed no apparent hepatic or renal toxicity, thus indicating excellent biocompatibility [129].

### 5.6. Calcium Carbonate-Based DDSs

Certain progress has been made in the development of nanomaterials based on calcium carbonate (CaCO_3_) for the delivery of anticancer drugs. CaCO_3_-based DDSs easily dissolve into Ca^2+^ and CO_2_ upon contact with the acidic environment, thus enabling pH-responsive controlled drug release in the tumor. Usually, calcium carbonate is employed for drug delivery in the form of vaterite due to a mesoporous structure of this polymorph modification. However, the dissolution rate of calcium carbonate at a pH above 6 and even at a pH above 5 is low, and this is a primary obstacle to using calcium carbonate for a such purpose. A probable solution is CaCO_3_ delivery into the endosomes of target cells, which can be obstructed by the carriers’ large size (above 300 nm) and their surface chemistry. Nevertheless, Zhiyu Zhang et al. developed a pH-responsive nanosystem based on calcium carbonate particles conjugated with a methoxy-poly(ethyleneglycol)-block-poly(L-glutamic acid) (mPEG-b-PGA) linker for the DOX delivery. The intravenous administration of particles to animals with the orthotopic osteosarcoma led to a slow gradual decrease of the DOX level in blood and a significant increase in the systemic circulation time, which is probably due to the mineralization of CaCO_3_ increasing the stability of nanoparticles, leading to reduced DOX leakage during circulation in vivo. CaNP/DOX carriers demonstrated a prominent inhibitory effect toward the K7 osteosarcoma growth as a result of excellent selective accumulation and release of DOX at the tumor site. The tumor suppression rate of free DOX and CaNP/DOX was 54.7% and 79.8%, respectively [267]. Alternatively, CaCO_3_ can be employed as a pH-responsive coating which solves both problems of CaCO_3_-based carriers: large size and low dissolution rate. In this way, hybrid carriers consist of manganese oxide (MnO_2_) particles modified with a pH-sensitive layer of CaCO3. DDS proposed by Daxiang Cui et al. is designed for the delivery of PD-L1-targeting siRNA, and the photosensitizer indocyanine green (ICG), which has a high potential in photodynamic and photothermal cancer therapy [268]. The systemic administration of these carriers to mice with a Lewis lung carcinoma demonstrated an enhanced antitumor response under laser irradiation and a decrease in tumor volume by nine times relative to the control group.

## 6. Current Challenges and Outlook

pH-responsive drug delivery is a promising technology since it enables site-specific cargo release by endogenous trigger. Apart from the general advantages of DDSs, such as a reduction of side effects, delivery of unstable and hydrophobic compounds, etc., the responsiveness of a carrier to endogenous pH as to a drug release trigger opens the prospects for improved tumor targeting and treatment of metastatic disease. However, there is still a multitude of challenges that are currently being resolved by the global research community. Although the initial concept of pH-responsive drug delivery was consistent, a number of problems revealed over the years significantly shifted requirements to drug carriers designed in the frame of this strategy.

Basic requirements for the carriers implied for systemic administration include size in the range of 20–200 nm and a negative charge to reduce their sequestration by immune cells during circulation in the blood flow. The analysis of recently published papers dedicated to metal-comprising carriers showed that the most omnipresent model drugs are doxorubicin, fluorouracil, and curcumin, and ζ-potential is a much more essential characteristic of DDS in terms of their loading capability than surface area, pores’ size, and volume. Moreover, it revealed the problem of the premature leakage of cargo from DDS with high DLC at a neutral pH, which can be a reason for systemic adverse effects, and formulated the demand for the improvement of carrier’s drug retention capacity. A meta-analysis of the data regarding different DDSs studied in vitro showed the importance of a high drug release rate and DLC for on-target cytotoxicity toward tumor cells required for successful therapy. Based on the analysis of IC_50_ of different DOX-loaded MOFs and metal NPs, we deduced that systems capable of quick release better suppress cancer cells’ metabolic activity in vitro compared to those releasing cargo over a long time. A high cargo-loading capacity is equally important for cancer cell suppression since it enables the achievement of the therapeutic concentration in the region of interest, which is also crucial for cancer therapy.

The other obstacle is that the difference in pH between healthy tissues and tumors of about 0.3–0.7 appears to be insufficient to provide a switchable release for 85% of carriers that rely on a release in response to a pH below 6 or even below 5.5. Moreover, the pHe within tumors is heterogeneous, and this represents a difficult task for pH-mediated tumor targeting strategy. These facts make the retrieval of novel pH-responsive materials and configurations capable of cargo release at pH values in the range of 6.2–6.8 an actual and challenging task.

The delivery of active substances via DDSs directly into endosomes of cancer cells is more effective in terms of pH responsiveness; however, it produces new challenges and requirements for pH-responsive carriers. The traffic of systemically administered carriers into endosomes of cancer cells is a highly complex multistage task. It becomes even more complex when taking into account that features facilitating the uptake of drug carriers by cancer cells impede features providing long-time circulation. Intracellular delivery requires the additional functionalization of a carrier to control complex processes of targeted internalization, release, and subsequent endosome escape of a drug into the cytoplasm to intracellular therapeutic targets. At the same time, systemically administered DDSs should provide prolonged circulation time in the blood flow to increase the probability of their accumulation in the diseased site and reduce the sequestration by the mononuclear phagocytic system, which, in its turn, impedes features required for effective uptake by cancer cells. These facts drive researchers to develop even more complex drug carriers that are capable of changing their properties in the tumor microenvironment, such as reversing their charge by protonation of carrier-imbedded compounds or PEG detachment from their surface to improve uptake, followed by controlled intra-endosomal release and endosomal escape. In this way, a carrier has to provide a cascade of functions both during circulation and after being extravasated into the tumor.

A particular problem relates to the slow diffusion of carriers from the blood flow into the parenchyma via the EPR effect in humans. The poor EPR effect nowadays seems to be the greatest challenge for the pH-responsive drug delivery concept since the penetration of carriers into the tumor interstitium is a pivotal step. Capable of long-term release, carriers are considered preferable for cancer therapy in light of their obstructed penetration into tumor tissues, and this pushes many researchers to focus their efforts on obtaining DDSs and providing sustainable drug release over a long time. Tremendous efforts have been made to improve the diffusion and accumulation of carriers in the tumor, as well as to develop alternative approaches circumventing mentioned restrictions. Thus, a number of approaches, including those enabling the disintegration of carriers into small moieties immediately upon entering the tumor or flash drug release in the vessels supplying the tumor, have been developed.

Despite substantial strides in this technology’s development, including significant success achieved in animal models, the results in clinical studies demonstrate poor survival. Although pH-responsive delivery undergoes a crisis of poor EPR effect in humans, it is still at the forefront of medical and material science. The current general trend toward increasing the complexity of the carrier’s structure, aiming to combine multiple functions and implement them in a cascade manner, as well as the development of alternative approaches, gives us hope that the currently developing systems will demonstrate success in clinical trials in the near future.

## 7. Conclusions

The field of pH-responsive drug delivery is intensively expanding given the growing number of publications per year. The technological evolution process is accompanied by new challenges and the need to correct the development vector under their influence. Therefore, existing-to-date trends in pH-responsive DDS design substantially differ from the initial concept. Thus, tumor treatment via systemically administered pH-sensitive DDSs implies their circulation in the blood flow, which results in a demand for their hiding from the immune system. The following step of pH-responsive drug delivery (tumor targeting) is characterized by the diversity of applied approaches, but the general vector of development can be defined as passive tumor targeting via the EPR effect enhanced by different methods of active targeting. The third step (drug release) gave birth to two contrary concepts: the one implying the release of cargo from pH-responsive DDSs at acidic pH common for tumor interstitium, and the other at the physiological pH of the blood. The concept of the drug release at acidic pH has passed a long evolutionary way and encountered multiple obstacles, including the insufficient difference in the pH of healthy and tumor tissues, inhomogeneity of pH over tumor interstitium and its poor blood supply, and discordance of features facilitating long-term circulation of DDSs and properties providing their high internalization potential by cancer cells. The concept of drug release at physiological pH has been proposed just recently and is at the beginning of its evolutionary way. The constant process of revealing new challenges and searching for their solutions ensures the development of drug delivery via pH-responsive DDSs. The increased focus of the research community in this field of medicine, as well as substantial strides in material science, enables us to believe that the next generation of pH-responsive DDSs will overcome discussed limits and provide an enhanced therapeutic effect.

## Figures and Tables

**Table 2:** $E_{rel}$, $E_{Ret}$, and DLC of different DDSs created in the frame of the quick drug release strategy. * Release value was estimated at pH 5.5; ** pH 5.2.

Active Substance	DDS Type	DDS Configuration	$E_{rel}\;(a.u.)$	$E_{Ret}\;(a.u.)$	DLC (%)	Ref.
Doxorubicin (DOX)	MOF (MIL)	DOX@ NH_2_-MIL-88B	2	-	7.4	[186]
	MOF (ZIF-90)	UC@mSiO_2_-RB@ZIF-O_2_-DOX-PEGFA	14.6 *	-	6	[150]
	MOF (ZIF-8)	DOX@ZIF-8	6.2	0.58	43.3	[174]
	MOF (ZIF)	UCMOFs@D@5	9	2.4	16.4	[151]
Fluorouracil (5-Fu)	MOF (ZIF)	UCMOFs@D@5	12	4	24.7	[151]
Curcumin (CUR)	MeO NPs	CUR-CS-ZnO	2.4 **	-	13	[152]

**Table 3:** $E_{rel}$, $E_{Ret}$, $t_{plat}$ and DLC of different DDSs created in the frame of the prolonged drug release strategy. * Release value was estimated at pH 5.5.

Active Substance	DDS Type	DDS Configuration	$E_{rel}\;(a.u.)$	$t_{plat}\;(h)$	$E_{Ret}\;(a.u.)$	DLC (%)	Ref.
Doxorubicin (DOX)	MOFs (ZIF-8)	DOX/HMS@ZIF	52.6	10	-	28	[182]
		BSA/DOX@ZIF	6.7	11	-	10	[160]
		DOX@ZIF-8@AS1411	34.2	20	12	-	[141]
		AuNCs@MOF-DOX	2.8	20	1	-	[212]
		ZIF-8@DOX@Organosilica	2.3	24	0.9	41.2	[174]
		ZIF-8@DOX	3.6	~72	1.3	-	[213]
		DOX@ZIF-8	3.3	50	1.3	-	[170]
		DOX@ZIF-8/Dex	2.8	68	1.7	63	
		H-ZIF-8/PDA-CD JNPs	3.2	30	1.1	-	[183]
		H-ZIF-8/PDA-CD JNPs + laser (808 nm, 1 W cm^−2^, 5 min)	2.7	-	-	-	
	MOFs (Cu-TCPP MOF)	Cu-TCPP-DOX	1.57	>60	0.75	-	[184]
	MOFs (graphene oxide/Cu (II)-porphyrin)	CuG1-DOX	2.8	60	1	45.7	
	MOFs (Fe-MOF)	DOX@FeMOF@PSS@MV-PAH@PSS	36.5	~24	12	88.4	[172]
	MOFs (γ-cyclodextrin-based MOF)	DOX/γ-CD-MOF	2.1	1	2.4	-	[185]
		DOX/GQDs@γ-CD-MOF	9.5	72	8	-	
		DOX/AS1411@PEGMA@GQDs@ γ-CD-MOF	4.8	96	2.4	-	
	MOFs (UIO-66)	UIO-66-NH_2_/PB/DOX	15.6	36	6	67.4	[171]
		Fe_3_O_4_@UIO-66-NH_2_/Graphdiyne/DOX	1.3	24	0.7	43.8	[167]
	MOFs (MIL-88B)	DOX@ NH_2_-MIL-88B-On-NH_2_-MIL-88B	1.8	10	-	14.4	[186]
	MeO NPs	MnO_2_ NPs@Keratin@DOX	1.4	24	0.7	8.7	[187]
Fluorouracil (5-Fu)	MOF	5-Fu@ [Zn_3_(BTC)_2_(Me)(H_2_O)_2_](MeOH)_13_	2.8	~48	1.3	34.32	[189]
	MOF	5-Fu@CS/Zn-MOF@GO	1.8	24	1.2	45	[162]
	MOF (ZIF-8)	5-FU@ZIF-8@Lf-TC	3.15	24	0.9	24.9 ± 1.4	[154]
Curcumin (CUR)	MeO NPs	CUR-CS-ZnO	2.25	10	-	13	[152]
		ZnO-PBA@CUR	31	36	4.8	35	[157]
		Fe_3_O_4_@Au-LA-CUR	4.6 *	6	1.3	-	[191]

**Table 4:** IC_50_ of drug-loaded DDSs presented as a concentration of capsulated substance (μg/mL) and an efficiency of cancer cell suppression (ECCS) of different DDSs after 24, 48, and 72 h. * Lipoic acid–curcumin.

Active Substance	Test Type	Cell Line	DDS Type	DDS Configuration	24 h		48 h		72 h		Ref.
					IC_50_	ECCS (a.u.)	IC_50_	ECCS (a.u.)	IC_50_	ECCS (a.u.)	
Doxorubicin (DOX)	MTT	MCF-7	MOFs (ZIF-8)	DOX/HMS@ZIF	~0.12	1.64	-	-	-	-	[182]
				BSA/DOX@ZIF	~0.04	1.48	-	-	~0.037	1.42	[160]
			MOF (NH_2_- MIL-88B (Fe))	DOX@FeMOF	<2.5	-	-	-	-	-	[172]
				DOX@FeMOF@PSS@MV-PAH@PSS	<2.5	-	-	-	-	-	
			MOFs (γ-cyclodextrin-based MOF)	γ-CD-MOF	>90	-	-	-	-	-	[185]
				GQDs@γ-CD-MOF	~34.7	-	-	-	-	-	
				DOX/AS1411@PEGMA@GQDs@ γ-CD-MOF	~15.76	-	-	-	-	-	
			MOF	DOX/CS/BioMOF	~2.45	1.3	-	-	-	-	[156]
			MOF (Cu (II)-porphyrin)	Cu(II)-porphyrin/ Graphene oxide-DOX	~4	1.38	-	-	-	-	[184]
		HeLa	MOFs (ZIF-8)	DOX@ZIF-8	~3	1.74	-	-	-	-	[141]
				DOX@ZIF-8@AS1411	~1.64	1.82	-	-	-	-	
				ZIF-8@DOX@Organosilica	~0.34	1.18	-	-	-	-	[174]
			MOF (ZIF-90)	UC@mSiO_2_@ZIF-DOX-PEGFA	>100	-	-	-	-	-	[150]
				UC@mSiO_2_-RB@ZIF-DOX-PEGFA + 808 nm laser irradiation	~36.7	1.6	-	-	-	-	
				UC@mSiO_2_-RB@ZIFO_2_-DOX-PEGFA + 808 nm laser irradiation	~15.5	2	-	-	-	-	
			MOFs (UIO-66)	Fe_3_O_4_@UIO-66-NH_2_/Graphdiyne/DOX	~20	0.62	~9.2	0.77	-	-	[167]
			MOF (Cu (II)-porphyrin)	Cu(II)-porphyrin/Graphene oxide-DOX	~14	1.16	-	-	-	-	[184]
		4T1	MOFs (ZIF-8)	AuNCs@MOF-DOX	~9.25	0.9	-	-	-	-	[212]
				AuNCs@MOF-DOX + 670 nm laser irradiation	~6	1.29	-	-	-	-	
			MOF (ZIF-90)	UC@mSiO_2_@ZIF-DOX-PEGFA	>100	-	-	-	-	-	[150]
				UC@mSiO_2_-RB@ZIF-DOX-PEGFA + 808 nm laser irradiation	~56.6	1.54	-	-	-	-	
				UC@mSiO_2_-RB@ZIFO_2_-DOX-PEGFA + 808 nm laser irradiation	~29.6	1.58	-	-	-	-	
			MOF	NiCo-PBA@DOX	>140	-	-	-	-	-	[142]
				NiCo-PBA@Tb^3+^@DOX	>140	-	-	-	-	-	
				NiCo-PBA@Tb^3+^@ PEGMA@DOX	~6.4	1.52	-	-	-	-	
				NiCo-PBA@Tb^3+^@ PEGMA@AS1411@DOX	~5.8	1.56	-	-	-	-	
		A549	MOF (NH2- MIL-88B (Fe))	DOX@FeMOF	<2.5	-	-	-	-	-	[172]
				DOX@FeMOF@PSS@MV-PAH@PSS	<2.5	-	-	-	-	-	
		HUVEC	MOF (Cu (II)-porphyrin)	Cu(II)-porphyrin/ Graphene oxide-DOX	>139.2	-	-	-	-	-	[184]
		NIH-3T3			>139.2	-	-	-	-	-	
	WST-8/CCK-8	MCF-7	MOF (ZIF-8)	DOX@ZIF-8/Dex	>10	-	-	-	-	-	[170]
		MCF-7/ADR			27.7	1.4	-	-	-	-	
		HeLa			>10	-	-	-	-	-	
			MOF	UCMOFs@Dox	~1.57	1	-	-	-	-	[151]
		4T1	MOF (NH2- MIL-88B)	DOX@NH_2_-MIL-88B	~3.5	-	-	-	-	-	[186]
				DOX@NH_2_-MIL-88B-On-NH_2_-MIL-88B	~2.8	-	-	-	-	-	
Fluorouracil (5-Fu)	MTT	A549	MOF	5-Fu/[Zn_3_(BTC)_2_(Me)(H_2_O)_2_](MeOH)_13_	~45.76	-	-	-	-	-	[189]
		HEK 293			>171.6	-	-	-	-	-	
		MDA-MB-231	MOF	5-Fu@CS/Zn-MOF@GO	-	-	-	-	~31.25	0.73	[162]
	WST-8/CCK-8	HeLa	MOF	UCMOFs@5-Fu	>83	-	-	-	-	-	[151]
Curcumin (CUR)	MTT	MCF-7	MeO NPs	ZnO-PBA@CUR	-	-	~9.58	1.45	-	-	[157]
		MCF-10a			-	-	>40	-	-	-	
		MDA-MB -231		CUR-CS-ZnO	-	-	~40	~0	-	-	[152]
		HEK 293			-	-	>100	-	-	-	
		Astrocytes		Fe_3_O_4_@Au-LA-CUR/GSH *	-	-	~97	0.64	-	-	[191]
		U87MG			-	-	~67.5	0.2	-	-	
	WST-8/CCK-8	A549	MOF (ZIF-L)	CUR@ZIF-L	~2.2	1.88	-	-	-	-	[190]
Camptothecin (CPT)	MTT	HeLa	MOF (ZIF-8)	CPT@ZIF-8@RGD	~3.3	1.87	-	-	-	-	[219]
			MOF (MIL)	MIL-101(Fe)-Suc-CPT	0.078 ± 0.016	-	-	-	-	-	[192]
				MIL-101(Fe)-Click-CPT	0.063 ± 0.015	-	-	-	-	-	
		3T3		MIL-101(Fe)-Suc-CPT	3.794 ± 0.459	-	-	-	-	-	
				MIL-101(Fe)-Click-CPT	6.393 ± 0.773	-	-	-	-	-	
		SH-SY5Y		MIL-101(Fe)-Suc-CPT	0.040 ± 3.2 × 10^−3^	-	-	-	-	-	
				MIL-101(Fe)-Click-CPT	0.029 ± 2.5 × 10^−3^	-	-	-	-	-	
Quercetin (Q)	MTT	MCF-7	MeO NPs	PBA-ZnO-Q	-	-	~7.36	1.3	-	-	[39]
				ZnO-Q	-	-	-	-	<1	-	[193]
		3T3-L1			-	-	-	-	>1	-	

## Data Availability

Data are available on request from the corresponding authors.
